# Preferential Enrichment of Enantiomer from Amino Acid Schiff Bases by Coordination Interaction and Crystallization

**DOI:** 10.3390/ma16020530

**Published:** 2023-01-05

**Authors:** Li Yan, Zhongkui Li, Xue Zhong, Jianxin Du, Yan Xiong, Shaochun Peng, Hui Li

**Affiliations:** 1 Key Laboratory of Cluster Science of Ministry of Education, School of Chemistry and Chemical Engineering, Beijing Institute of Technology, Beijing 102488, China; 2 Analysis & Testing Center, Liangxiang Campus, Beijing Institute of Technology, Liangxiang East Road, Beijing 102488, China

**Keywords:** preferential enrichment, amino acid, Schiff base, chirality

## Abstract

In this paper, preferential enrichment (PE) is described for three pairs of novel amino acid Schiff base Cu(II)/Cu(I) complexes. Single crystal X-ray diffraction indicated that **1-*S*/*R*** are one-dimensional coordination polymers (CPs) with helical structures, and **2-*S*/*R*** and **3-*S*/*R*** are one-dimensional CPs with auxiliary ligands. By tuning the pH, the solvent and second ligands, the **1**-***S*/*R***, **3-*S*/*R*** underwent polymorphic transitions, resulting in enantioselective liberation of excess enantiomers into solution, until the deposited crystals were slightly enriched with the opposite enantiomer, thereby successfully exhibiting PE. However, under the effects of Cu(II), the solvent and low pH, **2-*S*/*R*** did not exhibit PE and resulted in enrichment of racemic compounds, which was attributed to amino acid Schiff base chiral complex mechanisms of PE. The three pairs of Cu complex structures were characterized by UV-vis, MS and X-ray photoelectron spectroscopy (XPS). All chiral properties were studied by circular dichroism (CD) in the solid and liquid.

## 1. Introduction

Chirality, a universal feature that occurs through the whole process of generation and evolution of living organisms, is an important feature of biological organisms [1]. Amino acids are basic building blocks of all living organisms and they participate in all life activities. They typically occur in one of two possible enantiomeric forms, D forms and L forms [2,3,4], and they are also central sources of steric chiral carbon atoms. Salicylaldehyde-modified amino acids are among the most commonly used strategies for obtaining chiral ligands [5,6,7,8,9,10,11]. Their tridentate chelation coordination modes make it easier to interact with metal ions, which have antibacterial and antitumor activities after forming complexes.

**Scheme 1:**
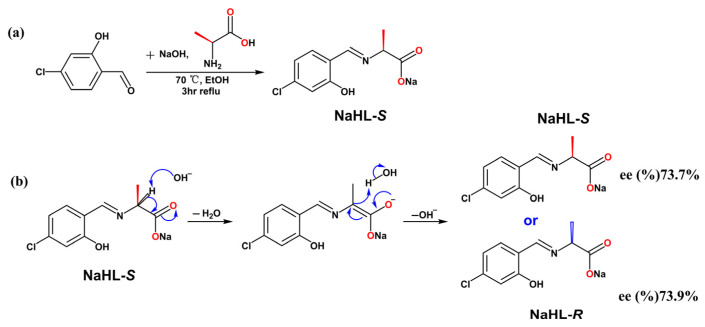
(**a**) Synthetic routes of **NaH-*S*** or **NaH-*R***; (**b**) racemization of the **NaHL**.

There are three main methods for obtaining chiral compounds: chiral source synthesis, chiral catalysis and chiral resolution. Chiral resolution has been the subject of considerable recent developments because it is economically viable, easy to operate and easy to realize industrial production. Preferential enrichment (PE) is an unusual symmetry-breaking spontaneous enantiomeric resolution phenomenon that applies to racemic crystals, the solvent-assisted solid-to-solid polymorphic transition of an initially formed metastable crystalline phase into another more stable one. Then, after partial crystal disintegration inside the crystal lattice, the enantioselective redissolution of excess enantiomers into the mother liquor leads to considerable enrichment of the same enantiomer until deposited crystals are slightly enriched with the opposite enantiomer [12,13,14]. The four conditions that are necessary for PE occurrence include: (i) Solubility difference; (ii) solid-to-solid polymorphic transition; (iii) partial crystal disintegration and (iv) deposition of nonracemic mixed crystals [15,16,17]. The PE phenomenon of amino acids has been reported. Uchida et al. theoretically analysed the mechanisms of PE. It is a spontaneous chiral amplification phenomenon that is caused by inherent chiral fluctuations in the racemic system, therefore, it is postulated that PE may be involved in formation of specificity of chiral substances (such as amino acids) in organisms [12]. Rajesh G. Gonnade et al. reported the PE of (DL)-phenylalanine/fumarate eutectic crystals under non-equilibrium conditions, proving that alanine racemic compounds did not exhibit PE but could exhibit this phenomenon with fumaric acid [18]. Since amino acids have multi-functions, they can easily be racemized by heating them in the presence of an aldehyde under neutral or weakly alkaline conditions (Scheme 1) [19,20,21]. Therefore, during the coordination process with metal ions, the salicylaldehyde-modified amino acids ligands easily exhibit racemization and the PE phenomenon. To the best our knowledge, the PE of amino acid Schiff bases have rarely been reported and should be investigated further.

In this work, the ligand (S, E)-2-((4-chloro-2-hydroxybenzylidene) amino) propanoate, **NaHL**) previously reported by our group [22,23,24] was studied in coordination with Cu(I) and Cu(II) ions by tuning the pH, the solvent and the second ligands. Three pairs of enantiomers of coordination complexes were obtained based on the PE principle, the different PE phenomena of complexes were observed during crystallization. The **1-*S*/*R***, **3-*S*/*R*** initially formed mixed crystals via the solvent-assisted solid-to-solid type polymorphic transition from kinetically formed metastable crystals to the thermodynamically stable ones. Subsequently, the partial crystal began to disintegrate, until the opposite enantiomers were preferentially enriched [17,18]. Under the induction of Cu(II), low pH, the solvent and the second ligands, the **2-*S*/*R*** failed to exhibit the PE phenomenon. The chiral properties of enantiomers were studied via solid and liquid circular dichroisms (CD).

## 2. Materials and Methods

### 2.1. Synthesis of Ligand 

The **NaHL-*S*** was prepared using 4-chloro-2-hydroxybenzaldehyde and ***L*** -alanine as raw materials (Scheme 1a). ***L*** -alanine (0.187 g, 5 mmol) and NaOH (0.2 g, 5 mmol) were added in ethanol (20 mL), the mixtures were stirred and refluxed for 1 h at 80 °C. The solution of 4-chloro-2-hydroxybenzaldehyde (0.7 g, 5 mmol, 10 mL) was added to the above solution, stirring and refluxing for another 3 h. The yellow solid was obtained (1.33 g, yield 89.8%). Anal. Calcd. (%) for C_10_H_9_ClNNaO_3_: C, 48.12; H, 3.63; N, 5.61. Found (%): C, 47.01; H, 3.93; N, 5.90. FT-IR (KBr cm^−1^): 1623 ν(C=O), 1139 ν(C-OH), 1576 ν(HC=N), 1290 ν(C-O), 728 ν(C-Cl).

The synthesis method of **NaHL-*R*** is similar to that of **NaHL-*S*** except that ***L*** -alanine is replaced with ***D*** -alanine. The yellow solid was obtained (1.43 g, yield 92.8%). Anal. Calcd. (%) for C_10_H_9_ClNNaO_3_: C, 48.12; H, 3.63; N, 5.61. Found (%): C, 47.41; H, 3.72; N, 5.54. FT-IR (KBr cm^−1^): 1629 ν(C=O), 1131 ν(C-OH), 1586 ν(HC=N), 1292 ν(C-O), 725 ν(C-Cl).

### 2.2. Synthesis of Complexes

#### 2.2.1. Synthesis of [Cu(L-S) (H_2_O)]_n_ (**1-*S***)

A mixture of Cu(NO_3_)_2_·6H_2_O (0.024 g, 0.1 mmol) and **NaHL**-**S** (0.024 g, 0.1 mmol) in H_2_O (3 mL) and ethanol (6 mL) was stirred for 30 min before filtration. The green stick single crystals suitable for single-crystal X-ray diffraction analysis were obtained after 8 days by solvent evaporation technique at room 89 m, 1447 temperature (yield: 43.26%). Anal. Calcd. (%) for C_10_H_8_ClCuNO_3_: C, 43.29; H, 3.96; N, 4.59. Found (%): C, 42.19; H, 3.56; N, 3.39 FT-IR (KBr cm^−1^): 3422.47 w, 1624.76 ν(C=O), 1588.71 ν(HC=N), 1518.21 w, 1427.06 m, 1372.09 w, 1335.25 m, 1288.41 ν(C-O), 1140.14 m, 1119.69 w, 1063.96 m, 978.61 w, 936.39 m, 889.48 w, 821.66 w, 861.58 m, 802.00 m, 769.43 w, 727.05 ν(C-Cl), 613.01 w, 585.51 w, 445.96 w, 421.02 w cm^−1^.

#### 2.2.2. Synthesis of [Cu(L-R) (H_2_O)]n (**1-*R***)

The synthesis method of **1-*R*** is similar to that of **1-*S*** except that **NaHL-*S*** is replaced with **NaHL-*R***. Additionally, the green stick single crystals were obtained after 8 days (yield: 49.17%). Anal. Calcd. (%) for C_10_H_8_ClCuNO_3_: C, 43.29; H, 3.96; N, 4.59. Found (%): C, 42.34; H, 3.09; N, 4.78. FT-IR (KBr cm^−1^): 3149.18 w, 1624.81 ν(C=O), 1588.96 ν(HC=N), 1512.09 w, 1447.40 w, 1457.52 w, 1425.31 w, 1399.36 s, 2335.35 w, 1297.17 w, 1288.59 ν(C-O), 1140.14 m, 1198.83 w, 1064.36 w, 978.78 w, 936.53 m, 861.70 w, 821.74 w, 802.09 m, 769.40 w, 727.19 ν(C-Cl), 613.10 w, 586.25 w, 512.40 w, 446.18 w cm^−1^.

#### 2.2.3. Synthesis of [Cu_2_(L-S)_2_ (4,4′-bipy)] (**2-*S***)

**NaHL-*S*** (0.024 g, 0.1 mmol) and CuSO_4_ (0.015 g, 0.1 mmol) were dissolved in methanol (2 mL) and acetonitrile (2 mL), and the mixture was stirred for 15 min at room temperature. A solution of 4,4′-bipyridine (0.015 g, 0.1 mmol) in methanol (1 mL) was added to the above solution and stirred for another 30 min before filtration. The green block single crystals suitable for single-crystal X-ray diffraction analysis were obtained after 1 day (yield: 76.33%). Anal. Calcd. (%) for C_15_H_12_ClCuN_2_O_3_: C, 51.39; H, 4.57; N, 7.05. Found (%): C, 51.76; H, 4.06; N, 6.83. FT-IR (KBr cm^−1^): 3451.31 m, 1631.99 ν(C=O), 1591.78 ν(HC=N), 1513.20 w, 1427.79 w, 1473.93 w, 1357.09 w, 1381.11 w, 1291.34 ν(C-O), 1264.68 w, 1225.74 w, 1191.97 w, 1131.73 w, 1118.33 w, 1070.55 w, 941.37 w, 864.15 w, 786.84 w, 723.19 ν(C-Cl), 617.20 w, 577.05 w, 617.20 w, 577.05 w, 464.26 w cm^−1^.

#### 2.2.4. Synthesis of [Cu_2_(L-R)_2_ (4,4′-bipy)] (**2-*R***)

The synthesis method of **2-*R*** is similar to that of **2-*S*** except that **NaHL-*S*** is replaced with **NaHL-*R***. The green block single crystals were obtained after 1 day (yield: 77.17%). Anal. Calcd. (%) for C_15_H_12_ClCuN_2_O_3_: C, 51.39, H, 4.57; N, 7.05. Found (%): C, 51.76; H, 4.06; N, 6.83. FT-IR (KBr cm^−1^): 3441.83 m, 1629.09 ν(C=O), 1612.72 w, 1589.27 ν(HC=N), 1511.51 w, 1458.06 w, 1439.22 w, 1384.15 w, 1359.02 w, 1299.93 ν(C-O), 1283.56 w, 1221.06 w, 1192.12 w, 1128.12 w, 1069.73 w, 933.32 w, 857.12 w, 817.25 w, 728.34 ν(C-Cl), 688.79 w, 643.945 w, 617.59 w cm^−1^.

#### 2.2.5. Synthesis of [Cu_2_(L-S)_2_(4,4′-bipy)]n (**3-*S***)

**NaHL-*S*** (0.012 g, 0.05 mmol) and CuCl (0.049 g, 0.05 mmol) were dissolved in methanol (2 mL) and DMF (2 mL), and the mixture was stirred for 15 min at room temperature. A solution of 4,4′-bipyridine (0.039 g, 0.05 mmol) in methanol (1 mL) was added and stirred for another 30 min before filtration. The green block single crystals suitable for single-crystal X-ray diffraction analysis were obtained after 1 day (yield: 75.73%). Anal. Calcd. (%) for C_30_H_24_Cl_2_Cu_2_N_4_O_6_: C, 49.54; H, 3.89; N, 7.45. Found (%): C, 48.76; H, 4.06; N, 6.83. FT-IR (KBr cm^−1^): 3139.84 m, 1632.57 ν(C=O), 1589.04 ν(HC=N), 1521.18 w, 1492.68 w, 1473.93 w, 1399.36 s, 1351.50 w, 1282.96 ν(C-O), 1225.76 w, 1195.25 w, 1137.84 w, 1035.33 w, 1012.05 w, 943.00 w, 929.25 w, 851.12 w, 842.52 w, 820.97 w, 800.40 w, 729.14 ν(C-Cl), 645.46 w, 613.46 w, 578.13 w, 469.98 w, 433.58 w cm^−1^.

#### 2.2.6. Synthesis of [Cu_2_(L-R)_2_(4,4′-bipy)]n (**3-*R***)

The synthesis method of **3-*R*** is similar to that of **3-*S*** except that **NaHL-*S*** is replaced with **NaHL-*R***. The green block single crystals were obtained after 1 day (yield: 87.17%). Anal. Calcd. (%) for C_30_H_24_Cl_2_Cu_2_N_4_O_6_: C, 49.54; H, 3.89; N, 7.45. Found (%): C, 47.98; H, 3.96; N, 7.83. FT-IR (KBr cm^−1^): 3131.29 m, 1636.69 ν(C=O), 1587.81 ν(HC=N), 1532.37 w, 1454.20 w, 1400.28 s, 1293.46 ν(C-O), 1225.76 w, 1195.24 w, 1138.22 w, 1076.28 w, 943.06 w, 929.93 w, 850.81 w, 799.60 w, 762.52 w, 729.05 ν(C-Cl), 613.53 w, 579.53 w, 579.56 w, 433.70 w cm^−1^.

### 2.3. Methods

The elemental analyses (C, H and N) were measured on an EA-3000 elemental analyser (Euro Vector, Italy). FT-IR spectra were recorded on Thermo IS 5 FT-IR spectrometers (Walthamm, MA, USA) in the range of 4000–400 cm^−1^. The PXRD data of the samples were measured on a Bruker D8 ADVANCE (Karlsruhe, Germany). Thermogravimetric analyses (TGA) were measured using a Japan Shimadzu DTG-60 H thermal analyser (Shimadzu, Tokyo, Japan). The UV-vis absorption spectra were collected on a UT-1950 spectrophotometer (Persee, Beijing, China) in the range of 200–800 nm. The liquid CD spectra were measured on a JASCO J-1500 spectropolarimeter (JASCO, Tokyo, Japan) in the range of 200–800 nm and the solid CD spectra were measured by KBr pellet (sample: KBr = 1: 200). ESI-MS spectra were measured using a Thermo Scientific (Walthamm, MA, USA) Q Exactive HF-X mass spectrometer equipped with an ESI source and in the positive ion mode from *m*/*z* 100 to 1600. HPLC were measured with Waters upc2 (Waters, Milford, MA, USA) Chiralpak IG using MeOH/CO_2_ = 20:80 with flow rate of 2.0 mL/min and detection at 214 nm. X-ray photoelectron spectra (XPS) were measured with a PHI 5000 Versaprobe III (ULVAC-PHI, Kanagawa, Japan) with monochromatized Al Kα-X-rays (Hv,1486.6 eV) operating at 150 W, and were calibrated by the BE of the C component (BE = 284.6 eV) coming from contamination carbon.

## 3. Results and Discussion

### 3.1. Crystal Structures

Single crystal diffraction data were measured on a Bruker DUO APEX II diffractometer using graphite monochromatic molybdenum Kα (λ = 0.71073 Å) radiation at room temperature, under the conditions of 45 KV and 30 mA. Diffraction data collection adopts *ω-2θ* scanning mode, and all scanned data are subjected to empirical absorption correction. The crystal structure was obtained by the SHELXL program [25,26]. All non-hydrogen atoms are determined and corrected by Fourier synthesis and refined anisotropically. Hydrogen atoms are in their theoretical positions and refined isotropically. The crystallographic data for **1-*S***, ***2*-*S*** and **3-*S*** are presented in Table 1, **1-*R***, ***2*-*R*** and **3-*R*** are presented in Table S1. The CCDC number from **1-*S*** to **3-*R*** are 2169253, 2169283, 2206482, 2206484, 2169254 and 2169256, respectively.

#### 3.1.1. Crystal Structures of **1-*S*** and **1-*R***

**Figure 1:**
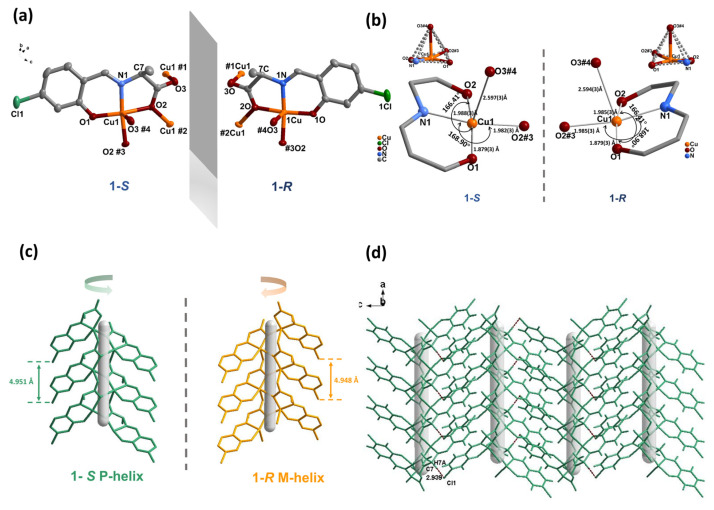
(**a**) Coordination environment presentation of **1-*S*** and **1-*R*** with 50% thermal ellipsoid probability (hydrogen atoms have been omitted for clarity); symmetry mode: #1:1/2 + x,1/2 − y, −z; #2: x, −1 + y, −1 + z; #3: −1 + x, −1 + y, −1 + z; #4: −3/2 + x, 1/2 − y, −z; (**b**) bond lengths and largest angles around the Cu(II) ion; (**c**) helical structures were formed via Cu1-O2-Cu1-O2 coordination modes of **1-*S***, **1-*R*** from *b* axis and P/M-helical structure with pitches of 4.951 Å and 4.948 Å; (**d**) 2D crystal structures of **1-*S*** were formed via non-classical H-bonding (C7-H7A…Cl1, 2.94, 3.669 (4) Å 132°).

Single crystal structure analysis revealed that **1**-***S*** and **1**-***R*** are a pair of enantiomers, and they both crystallize in the orthorhombic space group of *P2_1_2_1_2_1_*. Taking 1-***S*** as an example, the asymmetric unit is composed of one Cu (II) ion and one deprotonated ligand. The Cu(II) ion is five-coordinated and located in the centre of distorted square pyramidal coordination geometry of CuNO_4_. The N1, O1 and O2 from the ligand and O2#3 of the carboxyl group from the adjacent ligand occupy the equatorial positions, while the carboxylic acid oxygen atom (O3#4) from another ligand occupies the axis positions (Figure 1a). The **1**-***R*** exhibited a comparable coordination environment as **1**-***S***, except that the C7 carbon atom has an *R*-configuration (Figure S4). The ideal parameter for square-pyramidal geometry is α = *β* (the largest angles around the Cu(II) ion) = 180° with calculated τ = 0 [27,28,29]. The calculated geometry parameters τ of **1-*S*** and **1**-***R*** are 0.03 and 0.04, respectively. Therefore, they both exhibit a distorted square pyramidal coordination geometry (Figure 1b, Tables S3 and S4).

The asymmetric units were further extended into 1D chains along the *a* axis by the connection of O2#3 and O3#4 between adjacent ligands with Cu-Cu distance of 3.596(7) Å (Figure S5). A chiral Cu1–O2–Cu1–O2 helical chain was formed via coordination between Cu1 and O2. Structural analysis showed that helical chains in **1**-***S*** and **1**-***R*** exhibit a P-helical structure (right-hand) and an M-helical structure (left-hand) in space with pitches of 4.951 Å and 4.948 Å, respectively (Figure 1c and Figure S6). Additionally, fluorine atoms of the ligand moved towards the outside of the chain to form a hydrophobic surface. Non-classical hydrogen bonds (C7-H7A…Cl, 2.94 Å, 3.669 (4) Å, 132°) were formed between adjacent helical chains, which extended the 1D chain into higher 2D and 3D structures (Figure 1d, Figures S7 and S8).

#### 3.1.2. Crystal Structures of **2-*S*** and **2-*R***

**Figure 2:**
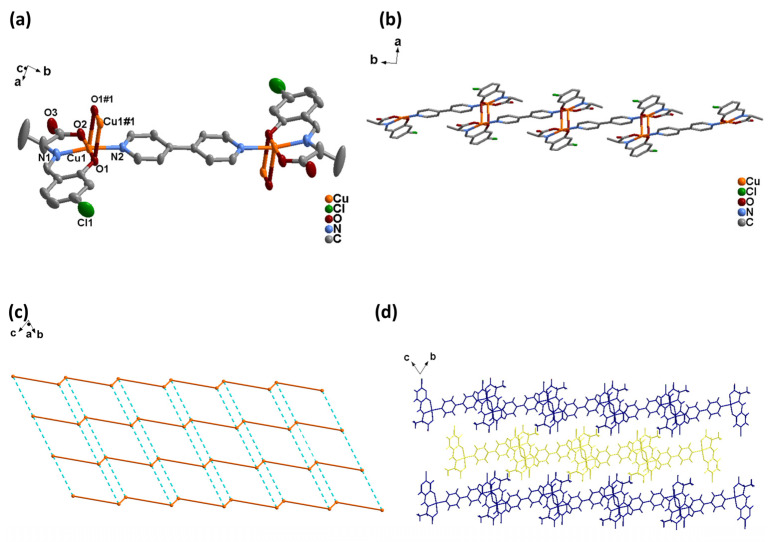
(**a**) Coordination environment presentation of **2-*S*** with 50% thermal ellipsoid probability (hydrogen atoms have been omitted for clarity); symmetry mode: #1: x, y, 1 + z; (**b**) 1D structure of **2**-***S*** as viewed from the *a* axis; (**c**) 2D topological structure of **2-*S*** as viewed from the *a* axis, the blue dotted line denotes the hydrogen bond; (**d**) the 3D supramolecular structure of **2*-S*** is packed in an ABAB form via van der Waals forces from the *b* axis.

Both **2-*S*** and **2-*R*** crystallized in the triclinic space group of *P*1. Taking **2-*S*** as an example, the asymmetric unit consisted of two Cu (II) ions, two deprotonated ligands and one 4,4′-bipyridine. The Cu(II) was five-coordinated and the geometry parameter τ was 0.18 for both **2-*S*** and **2-*R***, exhibiting a distorted square pyramidal geometry. The O1, O2 and N1 from ligand and N2 from 4,4′-bipyridine formed the equatorial plane, while O1#1 from the adjacent ligand occupied the axial position (Figure 2a and Figure S9, Table S5). The **2-*R*** exhibited a similar coordination environment as **2-*S*** (Figure S10). High flack parameters of 0.40 were observed in **2-*S*** (Table 1), proving the existence of enantiomers that come from ligands. Since **NaHL** was a racemic compound (Scheme 1b, Figure S12a,b), the other molecule in the unit cell, related by an inversion centre, has opposite chirality for both C9 and N1 (Figure S11) [30]. The ee (%) values were 38.1% and 1.56%, respectively, as determined by HPLC analysis (Figure S12c,d). Meanwhile, **2-*S*/*R*** pH was slightly higher than that of **1-*S*/*R*** (Table 2), thus, we postulated that under induction of Cu(II), 4,4′-bipyridine, the solvent and low pH environment, **2-*S*/*R*** did not exhibit the PE phenomenon [31,32], which produced the final racemic mixture [18].

Adjacent asymmetric units form 1D chain structures via Cu-O (phenolic hydroxyl) coordination bonds (Cu1-O1#1, O1-Cu1#1: 2.546(23) Å) (Figure 2b). The binuclear structure was observed in the 1D chain with a Cu-Cu distance of 3.392(7) Å. Non-conventional hydrogen bonds were formed between O3 from the carboxyl group and H14 from the benzene ring between adjacent chains (C14-H14A…O3, 2.37 Å, 3.322(5) Å, 162°), which assembled 1D chains into higher-dimensionality 2D structures (Figure 2c and Figure S13a). These layers were stacked together via van der Waals forces to form 3D supramolecular structures exhibiting ABAB packaging with slipping in spaces (Figure 2d and Figure S13c). Moreover, **2-*R*** exhibited similar stacking patterns as **2-*S*** (Figures S10c,d and S13b,d).

#### 3.1.3. Crystal Structures of **3-*S*** and **3-*R***

**Figure 3:**
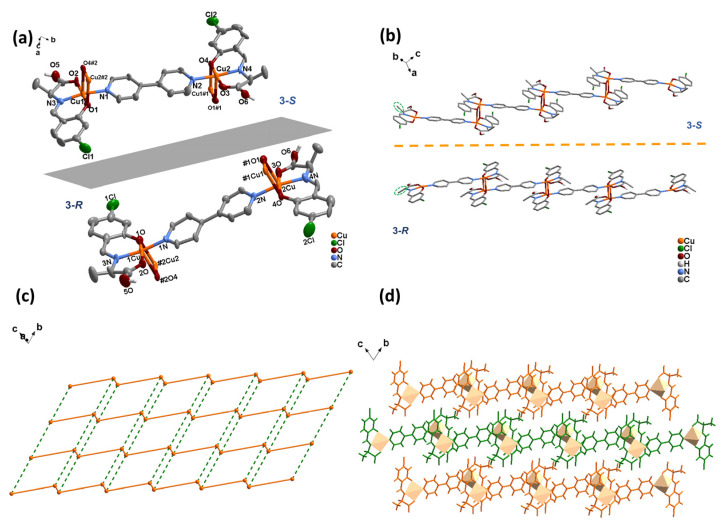
(**a**) Coordination environment diagram of **3-*S*/*R*** with 50% thermal ellipsoid probability (hydrogen atoms have been omitted for clarity); symmetry mode: #1: x, 2 + y, −1 + z; #2: x, y, 1 + z; (**b**) 1D structure of **3-*S*/*R*** as viewed from the *b* axis; (**c**) 2D topological structure of **3-*S*** as viewed from the *a* axis, the green dotted line denotes the hydrogen bond; (**d**) the 3D supramolecular structure of **3*-S*** is packed in an ABAB form via van der Waals forces from the *a* axis.

To verify the mechanisms of PE, **3-*S*** and **3-*R*** were synthesized using CuCl. They both crystallized in the triclinic space group *P1*. The asymmetric unit of **3-*S*** was comparable to that of **2-*S***, and it consisted of two Cu(I) ions, two deprotonated ligands, and one 4,4′-bipyridine (Figure 3a). The distance of Cu1-Cu2#2 was 3.364(3) Å. Furthermore, **3-*R*** exhibited a similar coordination environment as **3-*S***. Geometry parameters τ for **3-*S*** and **3-*R*** were 0.18 and 0.14, respectively, and the axial site of the Cu−O bond (Cu1-O4#2 distance:2.521(3) Å) was longer than the equatorial plane Cu−O bond distance (Cu1-O1, Cu1-O2:1.908(9) Å, 1.952(8) Å). Therefore, **3-*S*** and **3-*R*** exhibited distorted square pyramidal coordination geometries (Figure S14, Tables S7 and S8).

Under Cu(I) and higher pH conditions, the **3-S/*R*** exhibited the PE phenomenon during crystallization, with flack parameters of 0.00(4) and 0.13(5), respectively (Table 1 and Table S1). This implies that they are a pair of enantiomers, and it was observed that the **3-*S*** exhibited an *S*-configuration with the methyl up. The **3-*R*** exhibited an *R*-configuration with the methyl down (Figure 3b). Adjacent asymmetric units formed 1D chain structures via Cu-O (phenolic hydroxyl) bonds (Cu1-O4#2, Cu2-O1#1: 2.521(121) Å, 2.527(128) Å). Non-conventional hydrogen bonds were formed between adjacent asymmetric units with O from the carboxyl group on the ligand and H29 from the benzene ring (C29-H29A…O6, 2.32 Å,3.207 (2) Å, 161°), which assembled the 1D chain into a higher dimensionality 2D structure (Figure 3c and Figure S15). These layers were stacked together and expanded via van der Waals forces to form 3D supramolecular structures exhibiting an ABAB packaging with slipping (Figure 3d and Figure S16).

### 3.2. Thermal Stability and Powder X-ray Diffraction

**Figure 4:**
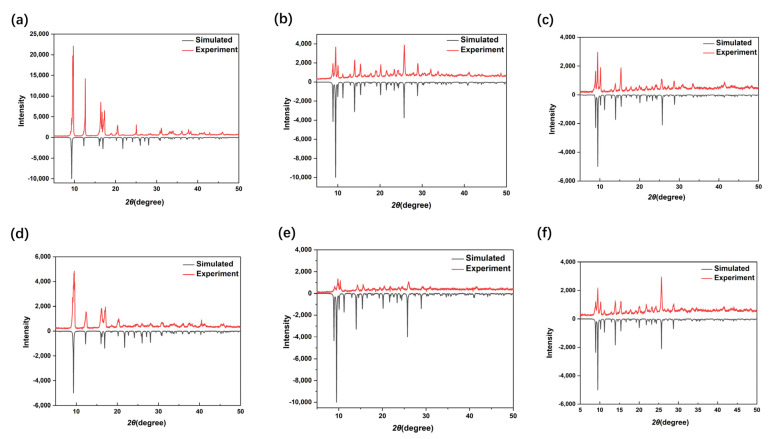
PXRD spectra of **1-*S*** (**a**), **1-*R*** (**d**), **2-*S*** (**b**), **2-*R*** (**e**), **3-*S*** (**c**) and **3-*R*** (**f**).

All complexes from **1-*S*** to **3-*R*** were obtained via the solvent evaporation technique at room temperature. High-quality single crystal structures of **1-*S*/*R***, **2-*S*/*R*** and **3-*S*/*R*** were obtained in ethanol-water, methanol-acetonitrile and methanol-DMF solutions, respectively. Findings from powder X-ray diffraction (PXRD) were highly consistent with simulated data of single crystals, indicating phase purity of samples (Figure 4). The TGA analysis showed that weight losses for **2-*S*/*R*** and **3-*S*/*R*** in the first stage were 4.1%/5.3% and 8%/10%, respectively, which were attributed to preferential loss of solvents, consistent with theoretical values. Decomposition temperatures of **1-*S*/*R***, **2-*S*/*R*** and **3-*S*/*R*** were 251 °C/248 °C, 221 °C/229 °C and 232 °C/234 °C, respectively, after which the frameworks of complexes began to collapse (Figure S17). 

### 3.3. UV-Vis Spectra of **NaHL**, and **1-S/R**, **2-S/R** and **3-S/R**

**Figure 5:**
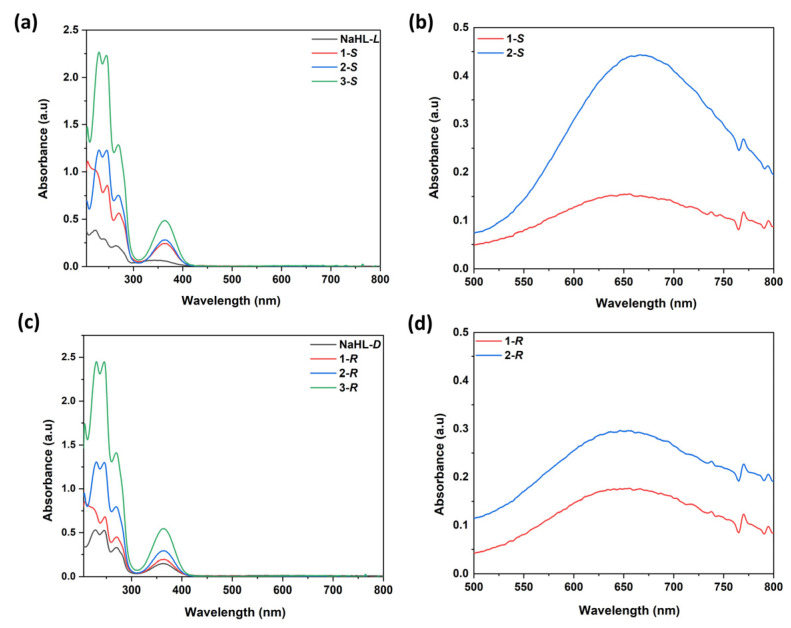
(**a**) UV-vis spectra of **NaHL-*S***, **1-*S***, **2*-S***, **3-*S*** in methanol with concentrations of 2.5 × 10^−5^ mol L^−1^; (**b**) UV-vis spectra of **1-*S***, **2-*S*** 500–800 nm in methanol with concentration of 1.0 × 10^−4^ mol L^−1^; (**c**) UV-vis spectra of **NaHL-*R***, **1-*R***, **2**-***R***, **3-*R*** in methanol with concentration of 2.5 × 10^−5^ mol L^−1^; (**d**) UV-vis spectra of **1-*R***, **1-*R*** 500–800 nm in methanol with concentration of 1.0 × 10^−4^ mol L^−1^.

The UV-vis spectra of **NaHL** and **1-*S*/*R***, **2-*S*/*R*** and **3-*S*/*R*** in methanol were recorded in the range of 200−800 nm. In Figure 5a,c, absorption peaks of **NaHL** at 222 nm, 242 nm and 265 nm were attributed to π→π* transitions of benzene rings. Absorption peaks at 350 nm were attributed to charge transfer transitions within the delocalized π-system in the molecular structure [33,34]. Enhanced absorption peaks for **1-*S*/*R***, **2-*S*/*R*** and **3-*S*/*R*** were observed in the range of 200–400 nm, and the intense high-energy band at 246 nm was attributed to π→π* intra-ligand charge transfer transitions (ILCT) [35,36]. The corresponding absorption peaks of complexes at 365 nm were strongly enhanced compared to that of **NaHL**, which was attributed to ligand to metal charge transfer (LMCT) transitions by strong interactions between metal ions and ligands [37,38,39,40]. This is consistent with X-ray diffraction crystallographic structures of those complexes. Due to d−d transitions of **1-*S*/*R*** and **2-*S*/*R***, very weak absorption peaks appeared between 600 and 700 nm in the UV-vis spectra (Figure 5b,d) [41,42,43].

### 3.4. Chirality and Chiral Recognition

**Figure 6:**
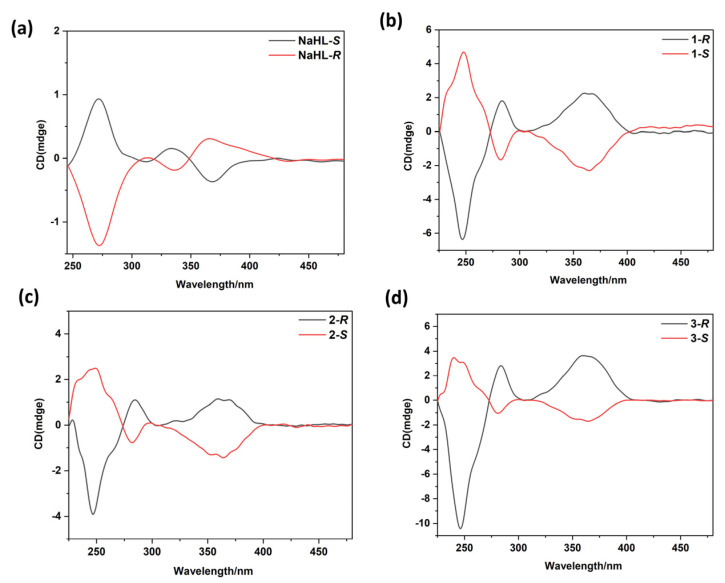
(**a**) CD spectra of **NaHL** in methanol with the concentration of 2.5 × 10^−5^ mol L^−1^. (**b**) CD spectra of **1-*S*** and 1-***R*** in methanol with the concentration of 2.5 × 10^−5^ mol L^−1^. (**c**) CD spectra of **2**-***S*** and **2-*R*** in methanol with the concentration of 2.5 × 10^−5^ mol L^−1^. (**d**) CD spectra of **3-*S*** and **3**-***R*** in methanol with the concentration of 2.5 × 10^−5^ mol L^−1^.

Considerable enantiomeric enrichment and polymorphic transitions have been correlated with chirality. Chiral properties of **NaHL** and **1-*S*/*R***, **2-*S*/*R*** and **3-*S*/*R*** were investigated via circular dichroism (CD) spectra in solution. The **NaHL-*S*** exhibited positive cotton effects at 271 nm and 335 nm, negative cotton effects at 315 nm and 369 nm, corresponding to absorption of Schiff-base ligands [44,45]. The **NaHL**-***R*** exhibited the opposite cotton effect at similar wavelengths (Figure 6a). Complexes 2-***S***/***R*** and 3-***S***/***R*** exhibited similar CD spectra with 1-***S***/***R*** (Figure 6b–d). The CD spectrum of **1**-***R*** exhibited opposite signals to those of **1-*S***, indicating that the two complexes are a pair of enantiomers (Figure 6b). Taking **1-S** as an example, a strong positive cotton peak was observed at 246 nm in **1*-S***, which was an obvious blue shift when compared with that of 283 nm in **NaHL** that was attributed to enhanced conjugation between ligands and metal ions. Negative cotton peaks were observed in the range of 300–400 nm, which was attributed to the LMCT process [5]. In the visible region of 500–800 nm, **1-*S*/*R*** and **2-*S*/*R*** exhibited weak CD signal peaks, which was attributed to d-d transitions in Cu(II) ions, corresponding to the UV-vis spectrum. Thus, the chiral information was transferred from the organic ligand to the metal ion (Figure S18) [46,47,48].

In the solid CD spectrum (Figure S19), **NaHL-*S*** exhibited a strong positive cotton effect at λ = 330 nm and a strong negative cotton effect at 284 nm. Compared with the liquid CD spectrum, signal peak of the π→π* transition absorption was weaker. This was attributed to abundant hydrogen-bond interactions between **NaHL** molecules in the solid, which inhibited π→π* transitions [49,50]. Furthermore, **1-*S*** showed positive cotton effects at 300 nm and negative cotton effects at 447 nm. Unlike the solution CD spectrum in the 500–800 nm region, CD signal peaks that were attributed to d-d transitions can be clearly observed. Compared to the CD spectra in solution, solid CD spectra of **1-*S*/*R***, **2-*S*/*R*** and **3-*S*/*R*** exhibited various differences. It is possible that interaction modes between components in the solution had changed due to solvent interference. Complexes from **1-*S*** to **3-*R*** between opposite cotton effects in solution and in solid CD spectra indicate the existence of a mirror-image relationship between complexes from **1-*S*** to **3-*R***, validating the presence of chiral enantiomers. Chirality of **1-*S*** to **3-*R*** was confirmed by single crystal structures.

### 3.5. ESI mass Spectrometry of **1-S/R**, **2-S/R** and **3-S/R**

The ESI-MS spectra was used to identify chemical species of **1-*S*/*R***, ***2-S*/*R*** and **3-S/*R*** in solutions. Molecular fragments in solutions of **1-*S*** and **1-*R*** were detected at 288.9558 *m*/*z*, which corresponded to the mononuclear component [Cu^2+^+L^2-^+H^+^] (calcd. 288.9562 *m*/*z*, Figure S20). In the methanol solution, **2-*S*** and **2-*R*** exists as a mononuclear structure ([Cu^2+^+L**^2-^**+bipy+H^+^], 445.0251 *m*/*z* and 445.0247 *m*/*z*, calcd. 445.0524 *m*/*z*) or binuclear structure ([2Cu^2+^+2L^2-^+bipy+H^+^], 732.9735 *m*/*z* and 732.9744 *m*/*z*, calcd. 732.9738 *m*/*z*) (Figure S21). Furthermore, **3-*S*** and **3-*R*** also existed as a mononuclear structure (445.0250 *m*/*z* and 445.0251 *m*/*z*, calcd. 445.0251 *m*/*z*) or binuclear structure (734.9718 *m*/*z* and 734.9715 *m*/*z*, calcd. 734.9895 *m*/*z*) in solution (Figure S22). Findings from ESI-MS spectra are consistent with X-ray crystallographic structures of complexes.

### 3.6. X-ray Photoelectron Spectroscopy (XPS) of **1-S/R**, **2-S/R** and **3-S/R**

**Figure 7:**
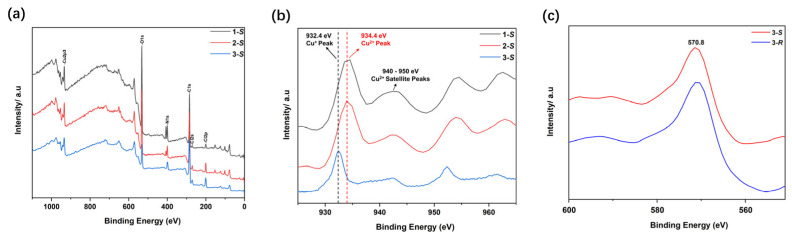
(**a**) Survey XPS spectra of **1-*S***, **2-*S*** and **3-*S***; (**b**) typical Cu 2p_3/2_ XPS spectra for **1-*S***, **2-*S*** and **3-*S***; (**c**) Cu^+^ LMM XPS spectra of **3-*S*** and **3-*R***.

To characterize the valence states of complexes, X-ray photoelectron spectroscopy (XPS) measurements were performed on **1-*S*/*R***, **2-*S*/*R*** and **3-*S*/*R***. Full-scan XPS spectra of complexes from **1-*S*** to **3-*R*** are shown in Figure 7a and Figure S23, which show the presence of Cu, Cl, N, O and C peaks. Table S2 summarizes the Cu 2p_3/2_, Cu 2p_1/2_, binding energy data of the complexes. The **1*-S***, **2*-S*** and **3*-S*** XPS spectra of Cu2p core level and Cu LMM (Auger spectra) are shown in Figure 7b,c. XPS indicates that the copper cation in **1*-S*** and **2*-S*** is bivalent. The **1*-S*** and **2*-S*** showed Cu 2p_3/2_ and Cu 2p_1/2_ peaks at 934.6 eV and 934.0 eV and 954.6 eV and 954.3 eV, respectively, while the satellite peaks from 940 to 945 eV were assigned to Cu^2+^ [51,52], For **3*-S*** the Cu 2p_3/2_ and Cu 2p_1/2_ binding energy peaks were observed at 932.4 eV and 954.2 eV, and the Cu LMM (Auger peak) at 570.1 eV can be clearly observed for that of Cu(I) [53,54], indicating the presence of Cu(I) centres [55,56]. Valence results of **1*-R***, **2*-R*** and **3*-R*** are consistent with those of **1*-******S***, **2*-S*** and **3*-S*** (Figure S23).

## 4. Conclusions

The PE phenomenon of the three pairs of amino acid Schiff base Cu complexes during crystallization have been assessed. The **1-*S***/***R*** and **3-*S*/*R*** exhibited polymorphic transitions during crystallization, resulting in enantioselective liberation of excess enantiomers into solution and preferential enrichment. However, under the effects of Cu(II), solvent and low pH, the **2-*S*/*R*** failed to exhibit a PE phenomenon, resulting in product racemic complexes. X-ray photoelectron spectroscopy (XPS) was used to characterize the Cu valence state. The solution and solid CD spectra revealed that intrinsic chiral activities of the amino acid Schiff base were transferred to the Cu centre. Based on the above findings, we postulated that PE can become a method for resolution of amino acid Schiff base chiral complexes, and the series of complexes can be applied in studies on resolution of chiral drugs and their synthetic intermediates in medicine and life sciences among others.

## Figures and Tables

**Table 1:** Crystallographic data for **1-*S***, **2-*S*** and **3-*S***.

Complex	1-*S*	2-*S*	3-*S*
Formula	C_10_H_8_ClCuNO_3_	C_30_H_25_Cl_2_Cu_2_N_4_O_6_	C_30_H_27_Cl_2_Cu_2_N_4_O_6_
*M* (mol^−1^)	289.16	732.91	734.51
*T* (K)	296(2)	296(2)	296(2)
*Crystal system*	Orthorhombic	Triclinic	Triclinic
*Space group*	*P2_1_2_1_2_1_*	*P1*	*P1*
*a* (Å)	4.9518(9)	8.0364(14)	8.0695(8)
*b* (Å)	10.8863(19)	10.2226(19)	10.1572(10)
*a* (Å)	19.221(3)	11.121(2)	10.9716(10)
*α* (°)	90	65.922(4)	66.809(2)
*β* (°)	90	79.246(4)	78.713(3)
*γ* (°)	90	86.931(4)	86.389(3)
*V* (Å^3^)	1036.2(3)	819.2(3)	810.52(14)
*Z*	4	1	1
*ρ(calculate)* (g·cm^−3^)	1.854	1.489	1.505
*F(000)*	580	372	372
*2θRange* (°)	4.238–56.55	4.078–49.912	4.11–51.994
*GOF on F^2^*	1.001	0.889	1.203
*R_int_*	0.0510	0.0707	0.1642
*Reflections collected*	12,869	6101	8536
*Independent reflections*	2581	5483	6467
*R_1_[I* > *2σ(I)]*	0.0311	0.0526	0.0589
*wR_2_[I* > *2σ(I)]*	0.0625	0.1144	0.1648
*R_1_* (all data)	0.0414	0.0935	0.0689
*wR_2_* (all data)	0.0669	0.124	0.1703
*Residuals* (e Å^−3^)	0.33/−0.27	0.41/−0.55	1.84/−1.36
*Flack parameter*	0.016(11)	0.40(5)	0.00(4)

**Table 2:** The **1-*S*/*R*** to **3-S/*R*** pH before and after synthesis.

	1-*S*	1-*R*	2-*S*	2-*R*	3-*S*	3-*R*
Before pH	2.62	2.75	4.41	3.65	8.92	9.02
After pH	2.7	2.76	4.74	4.23	9.33	9.19

## Data Availability

The data that support the findings of this study are available from the corresponding author upon reasonable request.

## Supplementary Materials

The following supporting information can be downloaded at: https://www.mdpi.com/article/10.3390/ma16020530/s1. See Supporting Information for related data.
