# Supplementary material for: Preferential Enrichment of Enantiomer from Amino Acid Schiff Bases by Coordination Interaction and Crystallization
Source: Materials (Basel). 2023 Jan 5;16(2):530. doi: 10.3390/ma16020530 (PMC9861923; doi:10.3390/ma16020530)
Supplement: Supplementary file 1 [file materials-16-00530-s001.zip › materials-2052115-supplementary.pdf]

Supporting Information

Li Yan [1,2], Zhongkui Li [1], Xue Zhong [1], Jianxin Du [2], Yan Xiong [2], Shaochun Peng [2] and Hui Li [1,*]

[1] Key Laboratory of Cluster Science of Ministry of Education, School of Chemistry and Chemical Engineering, Beijing Institute of Technology, Beijing 102488, China
[2] Analysis & Testing Center, Liangxiang Campus, Beijing Institute of Technology, Liangxiang East Road, Beijing 102488, China
* Correspondence: lihui@bit.edu.cn

Contents

Figure S1 FT-IR spectra of **NaHL-*S***, **1**-*S*, **2-*S*** and **3-*S***. 4

Figure S2 FT-IR spectra of **NaHL-*R***, **1**-*R*, **2-*R*** and **3-*R***. 4

Figure S3 Oak Ridge thermal ellipsoid plot (ORTEP) views of **1**-*S*, **1**-*R*, **2**-*S*, **2**-*R*, **3**-*S* and **3-*R*** with 50% thermal ellipsoid probability. 5

Figure S4 **1-*S*** (*S*-configuration) and **1**-***R*** (***R***- configuration). 5

Figure S5 The 1D chains of **1-*S***,**1-*R*** are connected by Cu-O viewed from *a* axis. 6

Figure S6 The helical structure via Cu1-O2-Cu1-O2 coordination modes of **1-*S***, **1-*R*** viewed from *b* axis. 6

Figure S7 2D crystal structure of **1-*R*** via on nonclassical H-bonding (C7-H7A…Cl1, 2.94,3.669 (4) Å 132°). 7

Figure S8 2D structure of **1-*S***, **1-*R*** viewed from *b* axis; 3D structure of **1-*S***,**1-*R*** viewed from *b* axis. 7

Figure S9 Coordination environment around Cu (II) in **2-*S***, **2-*R***. 8

Figure S10 Coordination environment diagram of **2-*R*** with 50% thermal ellipsoid probability (Hydrogen atoms have been omitted for clarity); Symmetry mode: #1: x, y, 1+z; 1D structure of **2-*R*** viewed from c axis; 2D layer structure of **2-*R*** formed by the 1D structure based on nonclassical H-bonding (C14-H14A…O3, 2.32 Å, 3.317(5) Å,162°); The 3D supramolecular structure of **2-*R*** is packed in ABA form via van der Waals forces from a axis. 8

Figure S11 The **2-*S*** and **2-*R*** have opposite chirality by reversing the center. 9

Figure S12 The **NaHL-*S*** and **NaHL-*R*** ee value was 73.7% and 73.9% determined by HPLC analysis. The **2-*S*** and **2-*R*** ee value was 38.1% and 1.56% determined by HPLC analysis. 9

Figure S13 2D layer structure of **2-*S*** and **2-*R*** formed by the 1D structure based on nonclassical H-bonding (C14-H14A…O3, 2.42 Å, 3.322(5) Å,162°). 3D structure of **2-*S*** and **2-*R*** viewed from b axis. 10

Figure S14 **3-*S*** and **3-*R*** the bond lengths and largest angles around the Cu (II) ion. 10

Figure S15 2D layer structure of **3-*R*** formed by the 1D structure based on nonclassical H-bonding (C29-H29A…O6, 2.31 Å,3.207 (2) Å,161°). 2D structure of **3-*S*** and **3-*R*** viewed from *c* axis. 11

Figure S16 The 3D supramolecular structure of **3-*S*** and **3-*R*** is packed in ABA form via van der Waals forces from *b* axis and *c* axis. 11

Figure S17 TG curves of **1-*S*** to **3-*R*** weight losses and collapse temperature. 12

Figure S18 CD spectra of **1-*S*** and **1-*R*** (1.0×10^{-4} mol l^{-1} in methanol); CD spectra of **2-*S*** and **2-*R*** (1.0×10^{-4} mol l^{-1} in methanol)........ 12

Figure S19 CD solid spectra of NaHL. CD solid spectra of **1-*S*** and **1-*R***. CD solid spectra of **2-*S*** and **2-*R***. CD solid spectra of **3-*S*** and **3-*R***. 13

Figure S20 ESI-Mass (ESI^+) spectrum of **NaHL-*S*** and **NaHL-*R*** in methanol. ESI-Mass (ESI^+) spectrum of **1-*S*** and **1-*R*** in methanol........ 14

Figure S21 ESI-Mass (ESI^+) spectrum of **NaHL-*S*** and **2-*S***. ESI-Mass (ESI^+) spectrum of **NaHL-*R*** and **2-*R*** in methanol........ 14

Figure S22 ESI-Mass (ESI^+) spectrum of **NaHL-*S*** and **NaHL-*R*** in methanol. ESI-Mass (ESI^+) spectrum of **3-*S*** and **3-*R*** exists as a single ligand or dual ligand structure in methanol. 15

Figure S23 Surveys XPS spectra of **1-*R***, **2-*R*** and **3-*R***; Typical Cu $2p_{3/2}$ XPS spectra for **1-*R***, **2-*R*** and **3-*R***........ 15

Table S1 Crystallographic Data for **1-*R***, **2-*R*** and **3-*R***. 16

Table S2 Cu 2p3/2, Cu 2p1/2 Binding Energies from XPS Measurements 16

Table S3 Selected bond distances (Å) and angles (°) for **1-*S***........ 17

Table S4. Selected bond distances (Å) and angles (°) for **1-*R***. 18

Table S5 Selected bond distances (Å) and angles (°) for **2-*S***........ 19

Table S6 Selected bond distances (Å) and angles (°) for **2-*R***. 20

Table S7 Selected bond distances (Å) and angles (°) for **3-*S***........ 21

Table S8 Selected bond distances (Å) and angles (°) for **3-*R***. 23

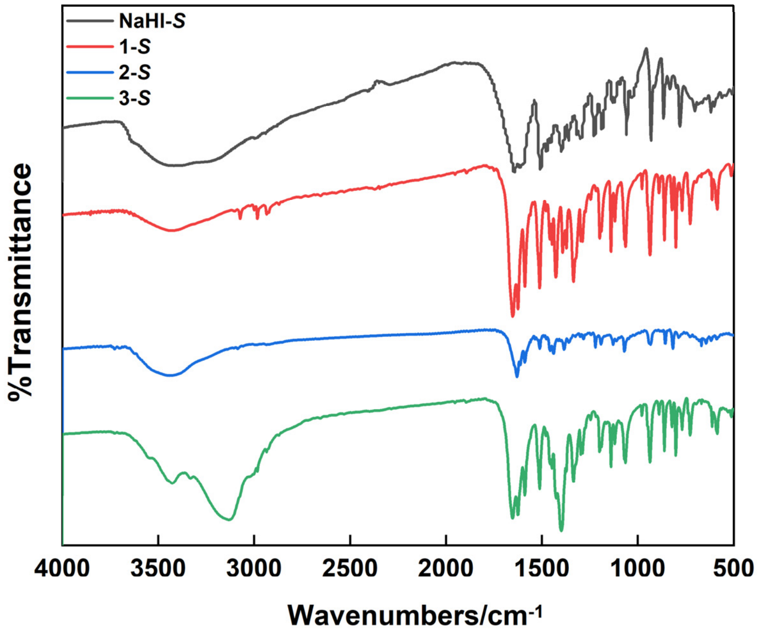

Figure S1 FT-IR spectra of **NaHL-*S***, **1-*S***, **2-*S*** and **3-*S***.

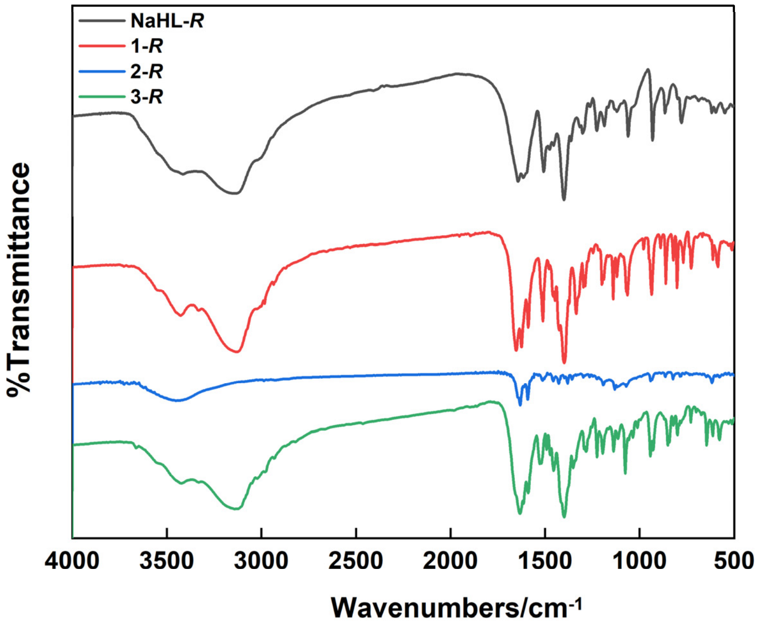

Figure S2 FT-IR spectra of **NaHL-*R***, **1-*R***, **2-*R*** and **3-*R***.

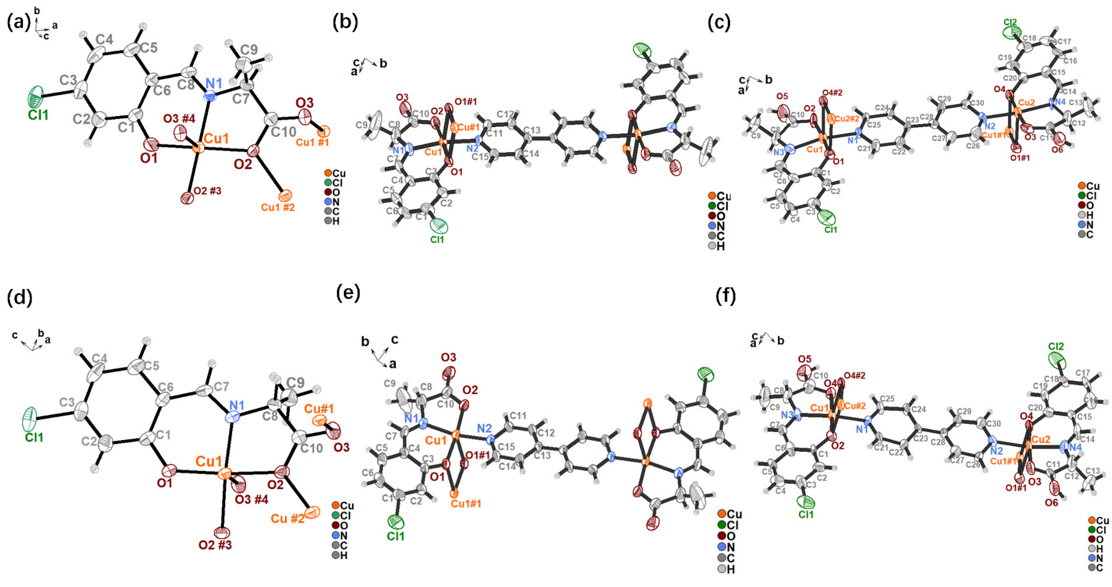

Figure S3 Oak Ridge thermal ellipsoid plot (ORTEP) views of **1**-*S* (a), **1**-***R*** (d), **2**-*S* (b), **2**-***R*** (e), **3**-*S* (c) and **3**-***R*** (f) with 50% thermal ellipsoid probability.

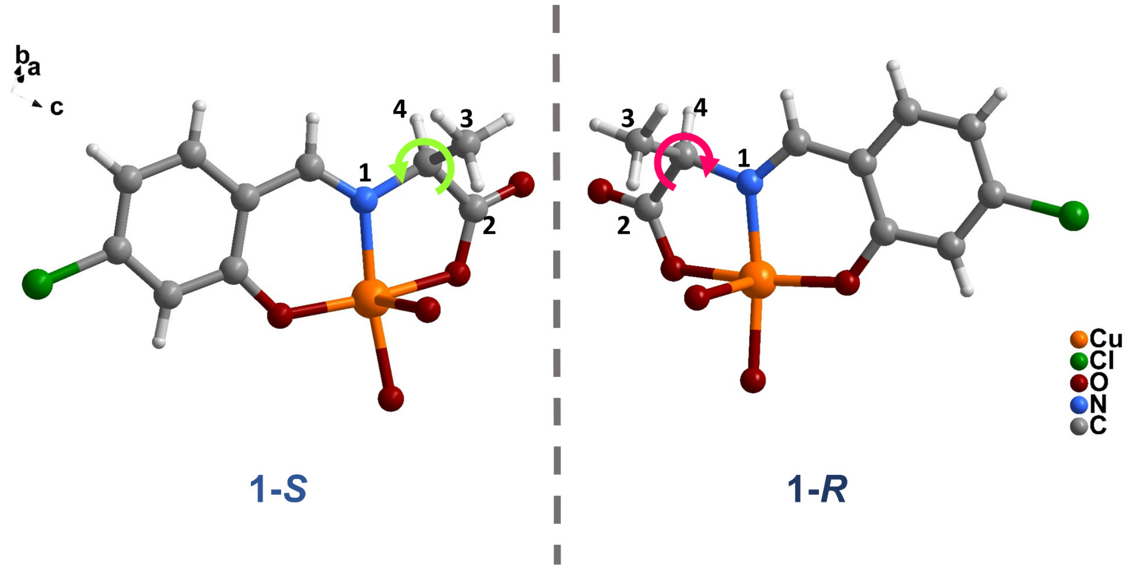

Figure S4 **1**-*S* (*S*-configuration) and **1**-***R*** (***R***- configuration).

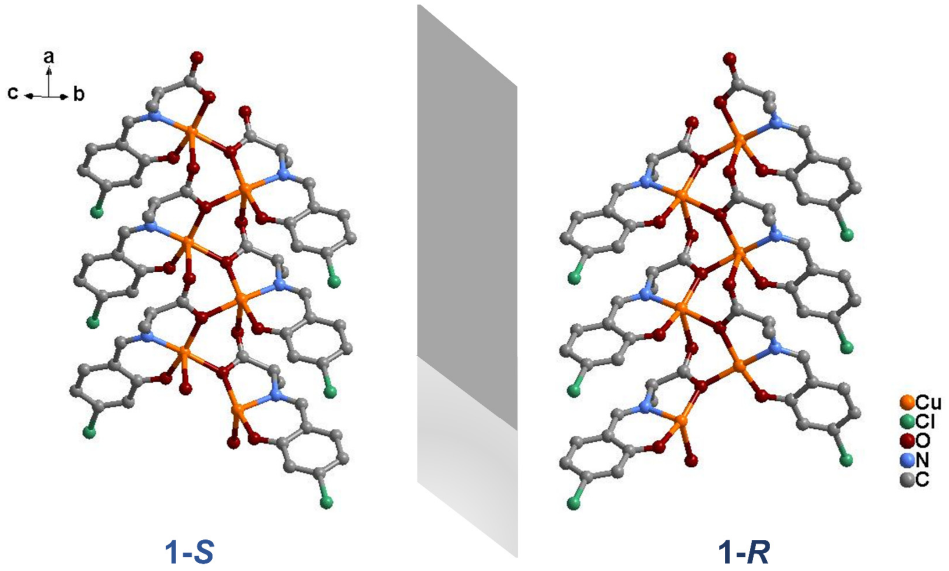

Figure S5 The 1D chains of **1-*S***,**1-*R*** are connected by Cu-O viewed from *a* axis.

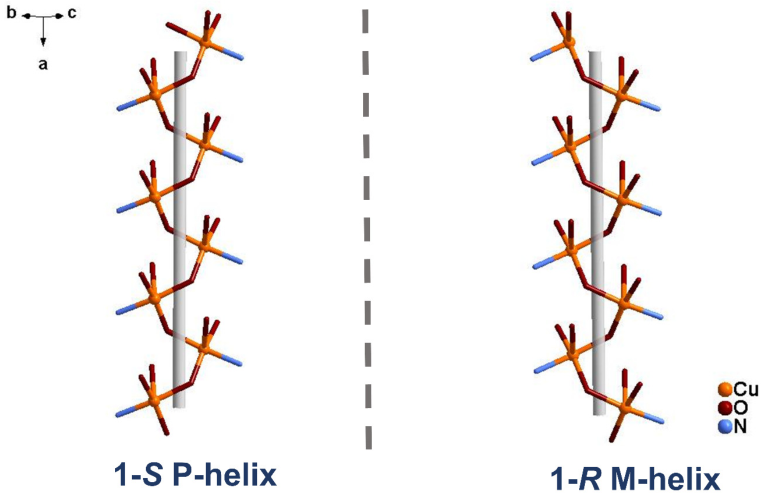

Figure S6 The helical structure via Cu1-O2-Cu1-O2 coordination modes of **1-*S***, **1-*R*** viewed from *b* axis.

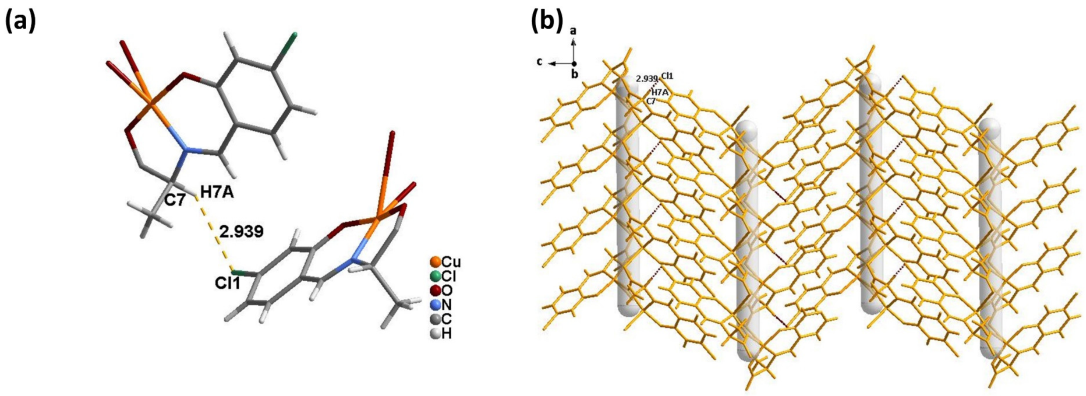

Figure S7 2D crystal structure of **1-*R*** via on nonclassical H-bonding (C7-H7A…Cl1, 2.94,3.669 (4) Å 132°).

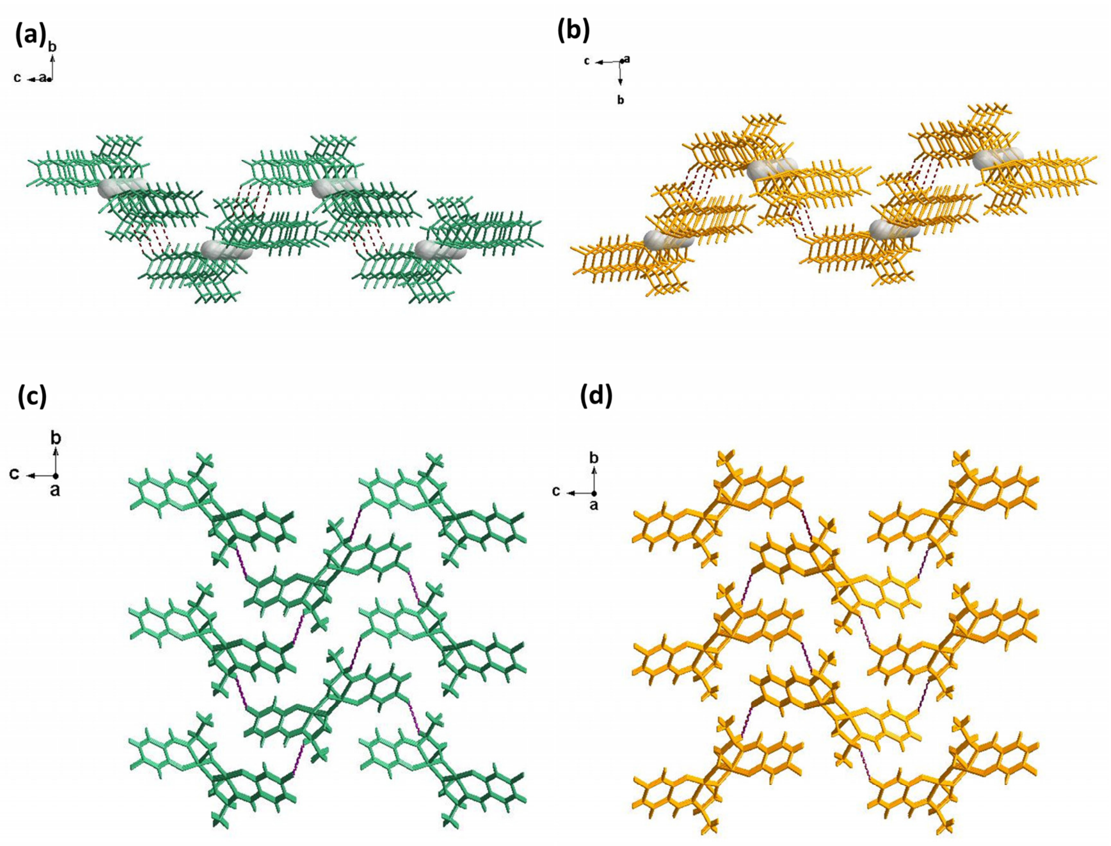

Figure S8 2D structure of **1-*S***, **1-*R*** viewed from *b* axis (a) and (b); 3D structure of **1-*S***,**1-*R*** viewed from *b* axis (c) and (d).

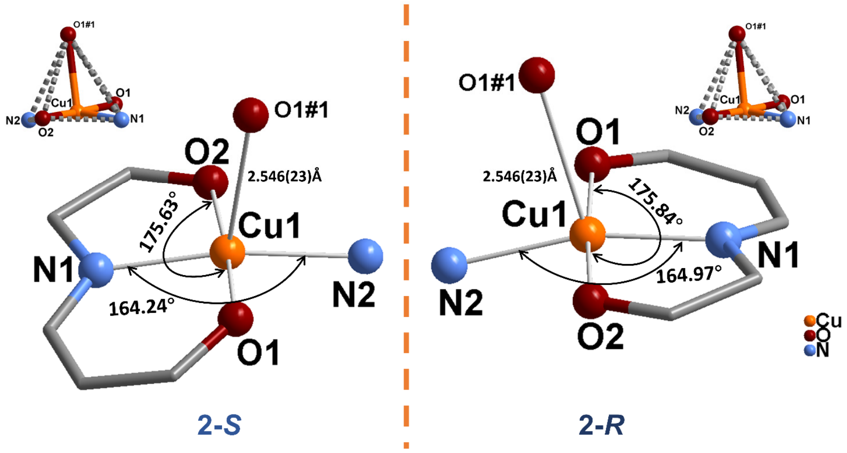

Figure S9 Coordination environment around Cu (II) in **2-*S***, **2-*R***.

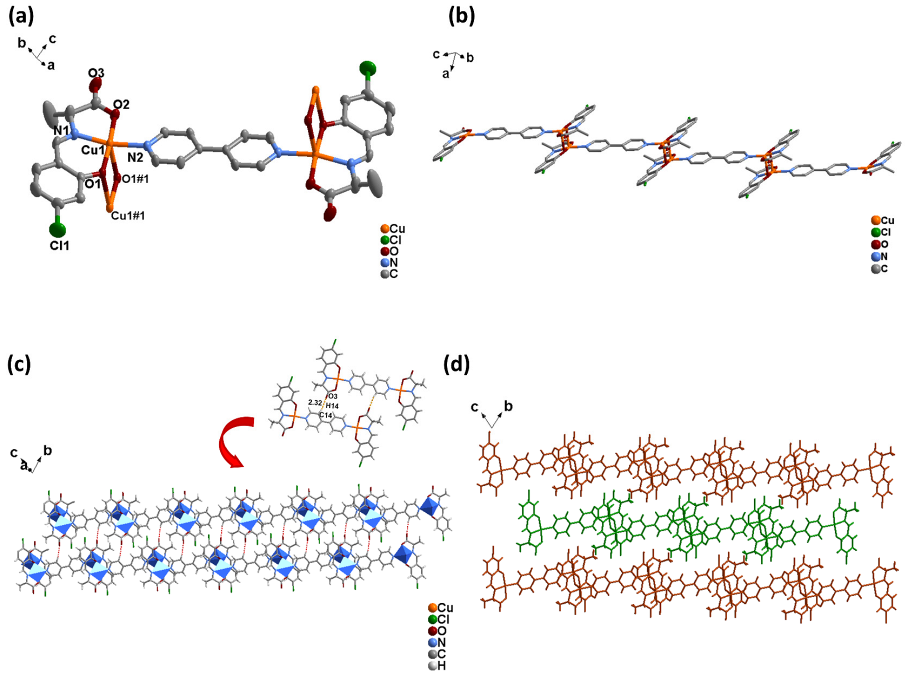

Figure S10 (a) Coordination environment diagram of **2-*R*** with 50% thermal ellipsoid probability (Hydrogen atoms have been omitted for clarity); Symmetry mode: #1: x, y, 1+z; (b) 1D structure of **2-*R*** viewed from c axis; (c) 2D layer structure of **2-*R*** formed by the 1D structure based on nonclassical H-bonding (C14-H14A…O3, 2.32 Å, 3.317(5) Å,162°); (d) The 3D supramolecular structure of **2-*R*** is packed in ABA form via van der Waals forces from a axis.

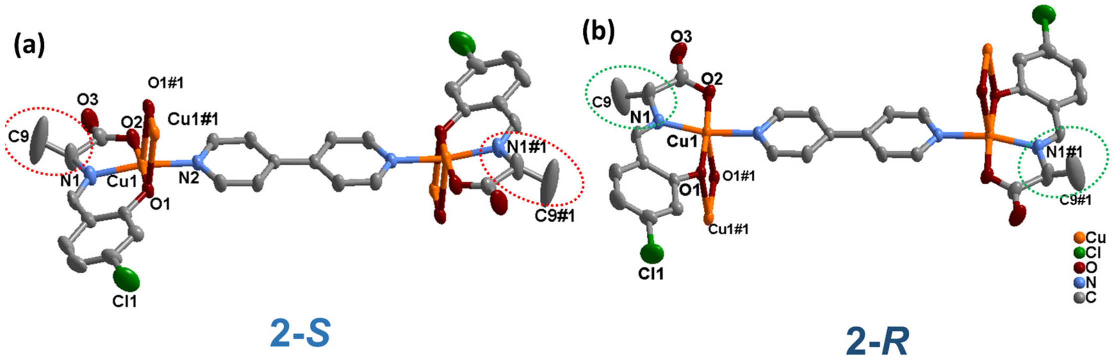

Figure S11 The **2-*S*** (a) and **2-*R*** (b) have opposite chirality by reversing the center.

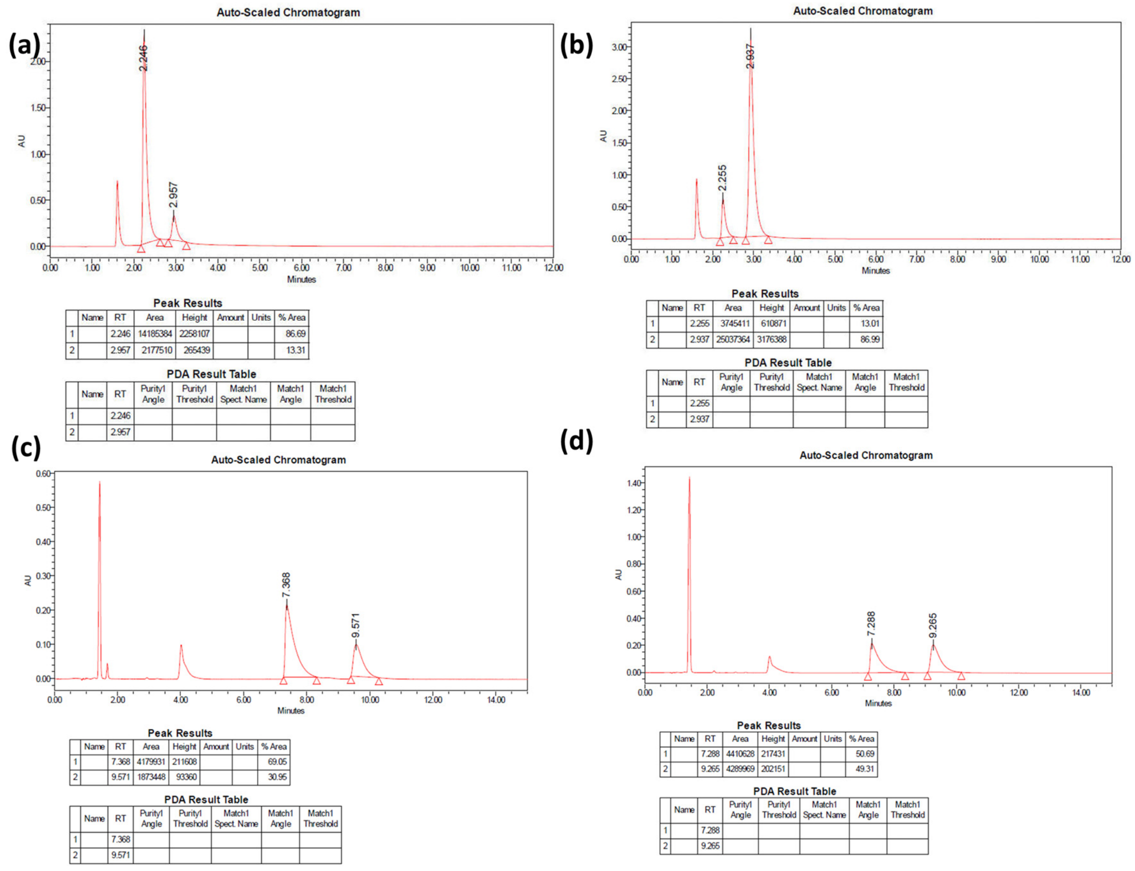

Figure S12 The (a) **NaHL-*S*** and (b) **NaHL-*R*** ee value was 73.7% and 73.9% determined by HPLC analysis. The (c) **2-*S*** and (d) **2-*R*** ee value was 38.1% and 1.56% determined by HPLC analysis.

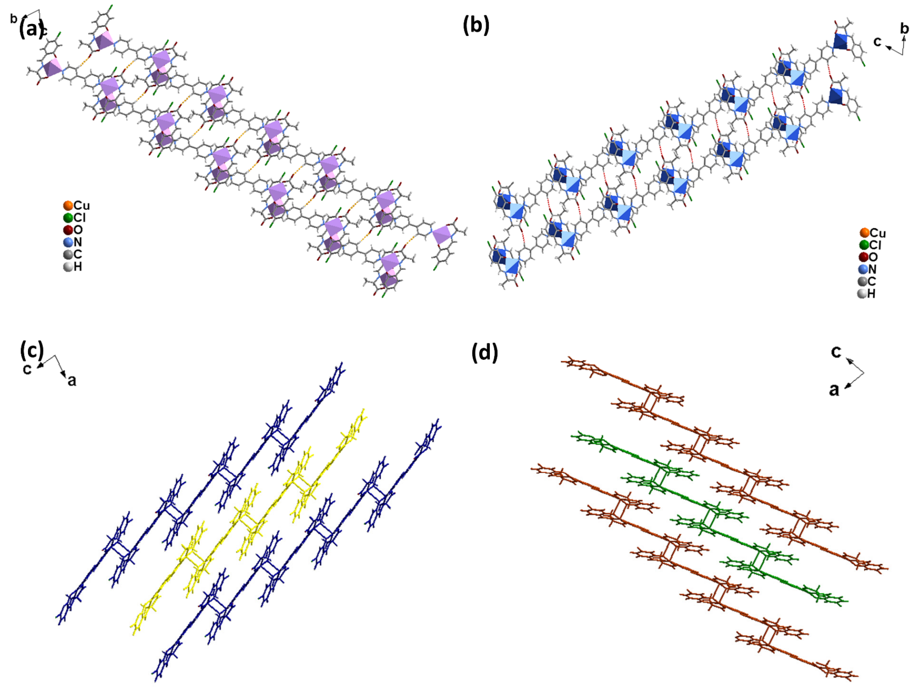

Figure S13 (a) 2D layer structure of **2-*S*** (a) and **2-*R*** (b) formed by the 1D structure based on nonclassical H-bonding (C14-H14A…O3, 2.42 Å, 3.322(5) Å,162°). 3D structure of **2-*S*** and **2-*R*** viewed from b axis (b) and (c).

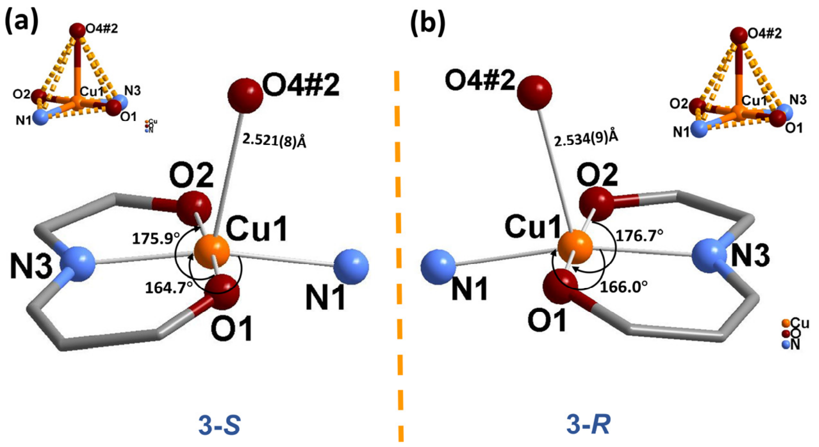

Figure S14 **3-*S*** (a) and **3-*R*** (b) the bond lengths and largest angles around the Cu (II) ion

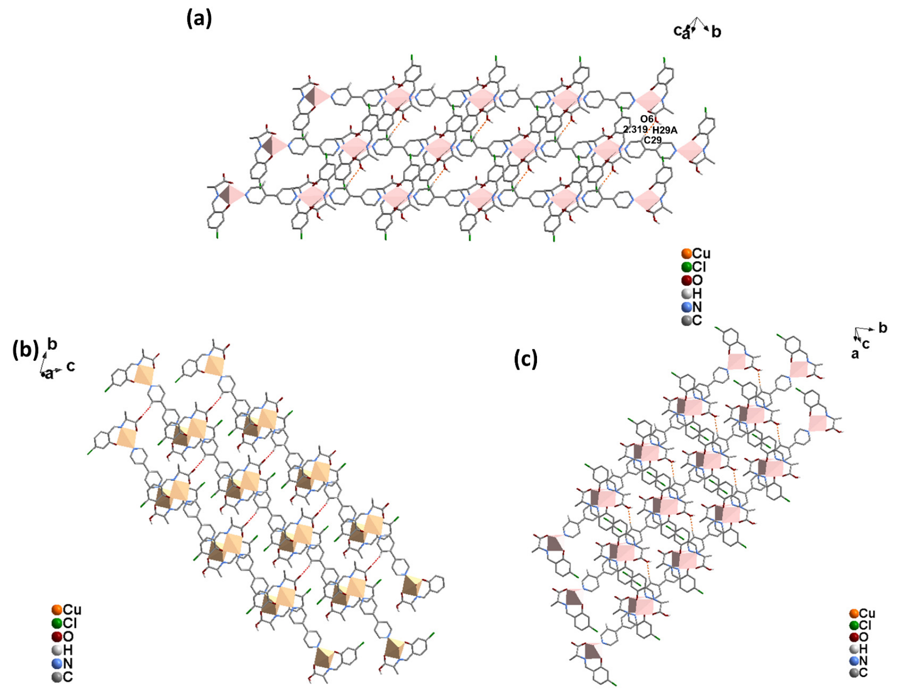

Figure S15 (a) 2D layer structure of **3-*R*** formed by the 1D structure based on nonclassical H-bonding (C29-H29A…O6, 2.31 Å,3.207 (2) Å,161°). 2D structure of **3-*S*** and **3-*R*** viewed from *c* axis (b) and (c).

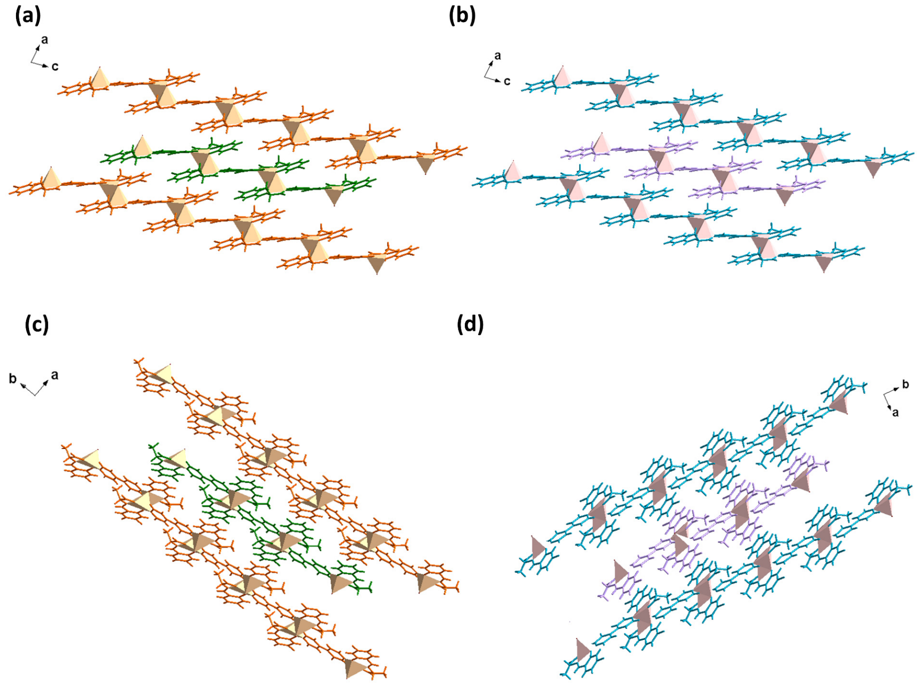

Figure S16 The 3D supramolecular structure of **3-*S*** and **3-*R*** is packed in ABA form via van der Waals forces from *b* axis(a), (b) and *c* axis (c), (d).

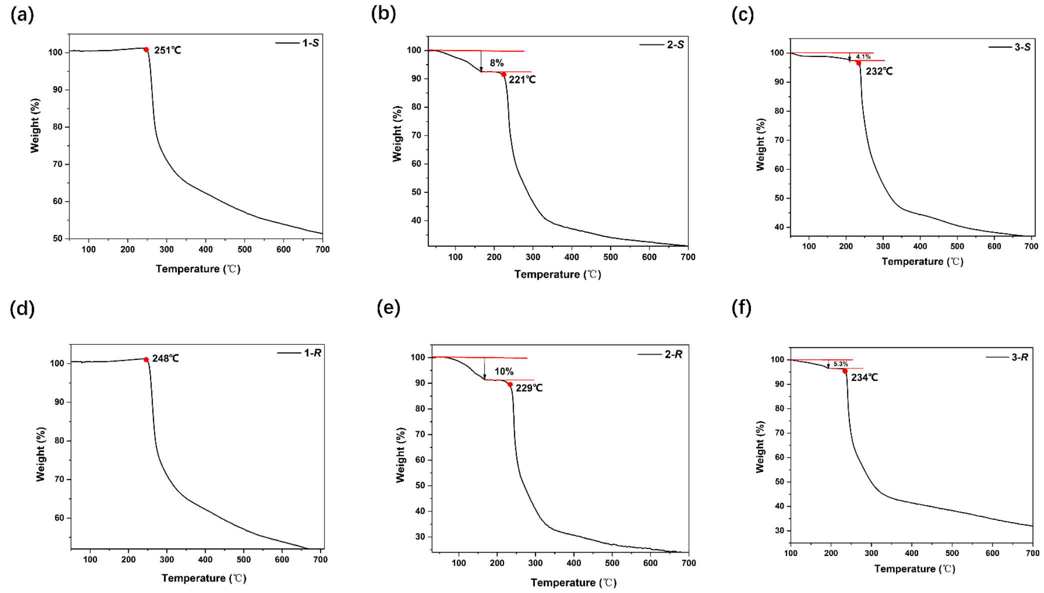

Figure S17 TG curves of **1-*S*** to **3-*R*** weight losses and collapse temperature, decomposition temperatures of **1-*S*/*R*** were 251 °C/248 °C (a and d), **2-*S*/*R*** were 221 °C/229 (b and e) and **3-*S*/*R*** were 232 °C/234 °C (c and f).

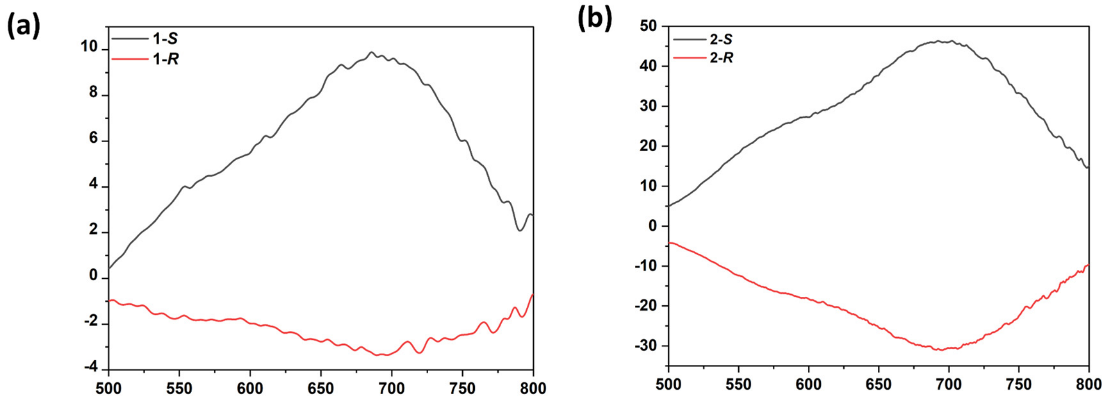

Figure S18 (a) CD spectra of **1-*S*** and **1-*R*** (1.0×10^{-4} mol l^{-1} in methanol); (b) CD spectra of **2-*S*** and **2-*R*** (1.0×10^{-4} mol l^{-1} in methanol).

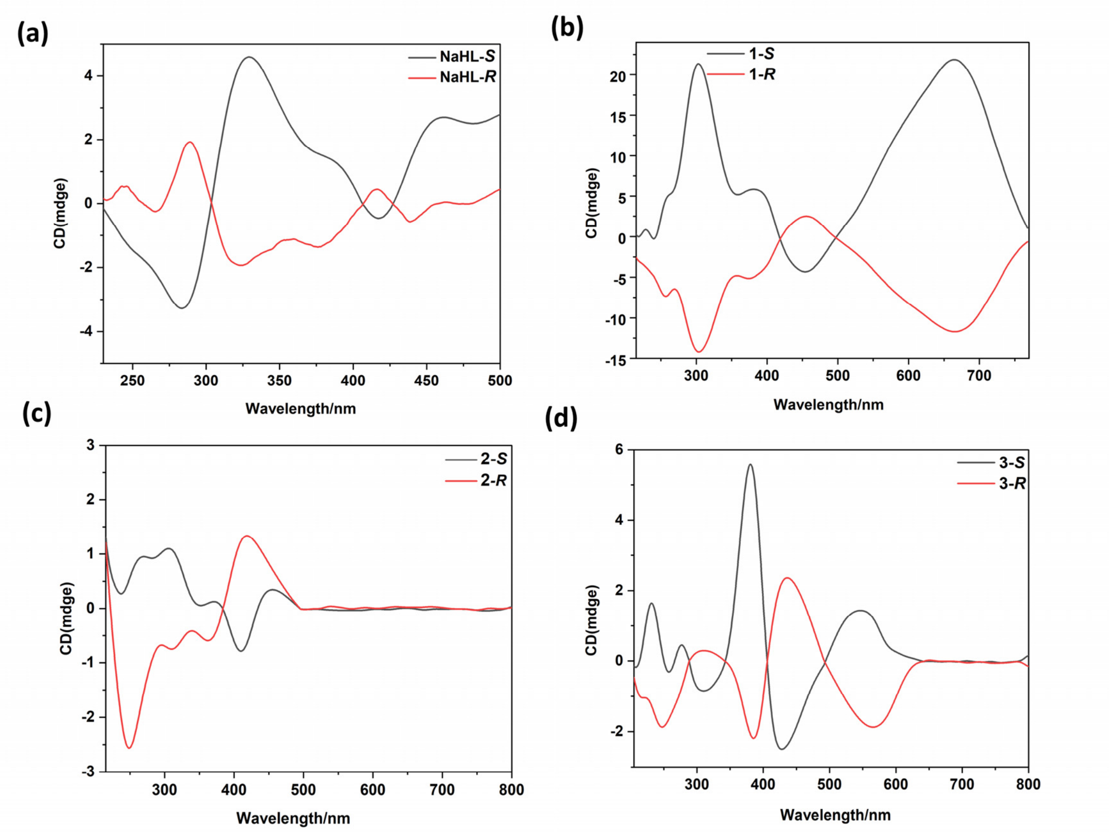

Figure S19 (a) CD solid spectra of NaHL. (b) CD solid spectra of **1-*S*** and **1-*R***. (c) CD solid spectra of **2-*S*** and **2-*R***. (d) CD solid spectra of **3-*S*** and **3-*R***.

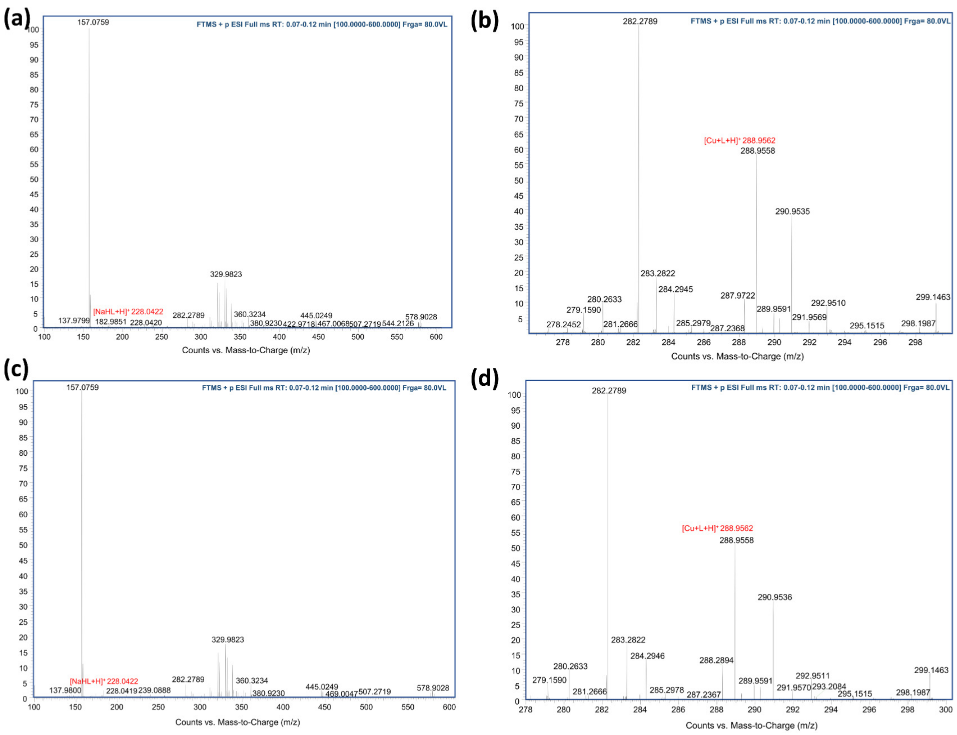

Figure S20 (a) and (c) ESI-Mass (ESI$^+$) spectrum of **NaHL-*S*** and **NaHL-*R*** in methanol. (b) and (d) ESI-Mass (ESI$^+$) spectrum of **1-*S*** and **1-*R*** in methanol.

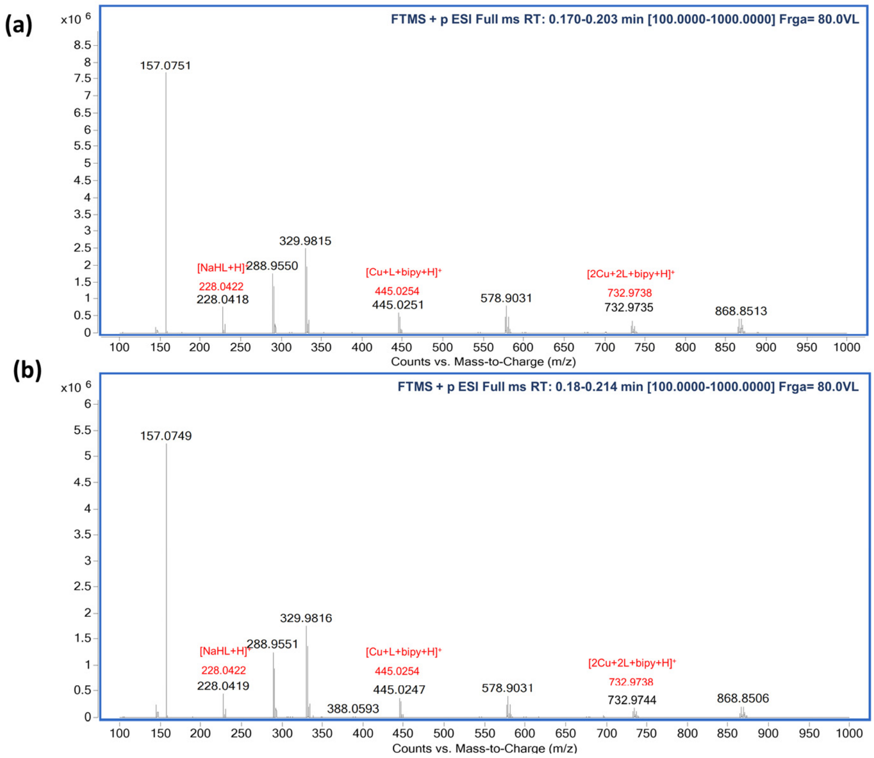

Figure S21 (a) ESI-Mass (ESI$^+$) spectrum of **NaHL-*S*** and **2-*S***. (b) ESI-Mass (ESI$^+$) spectrum of **NaHL-*R*** and **2-*R*** in methanol.

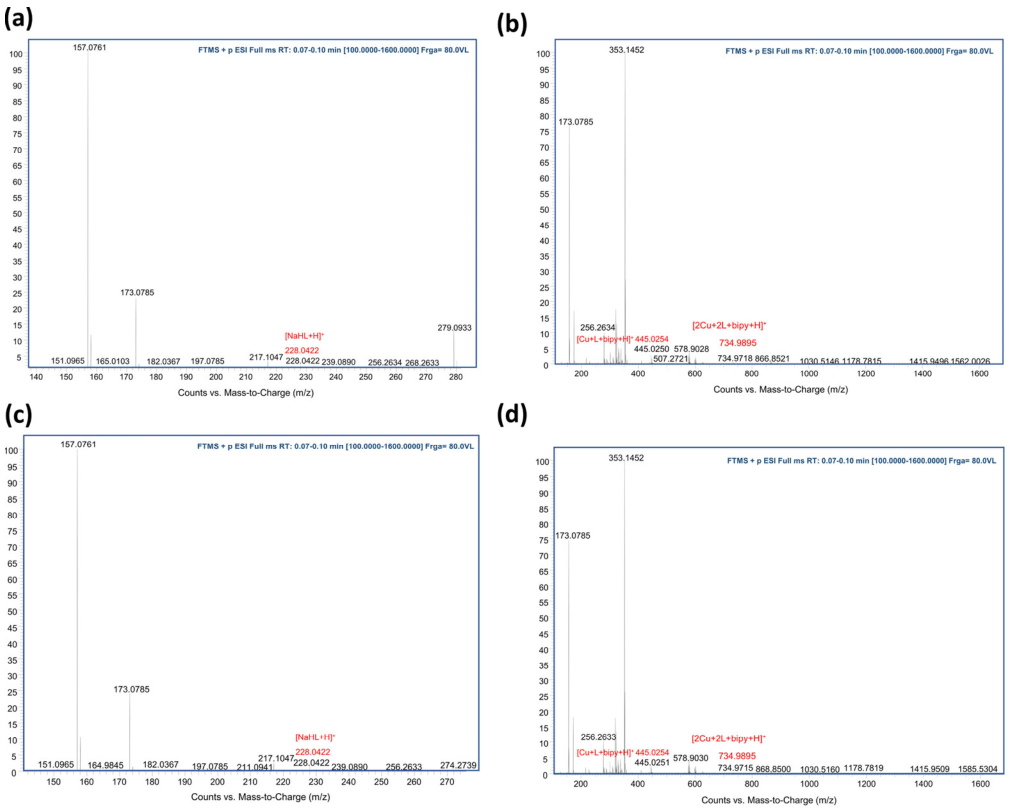

Figure S22 (a) and (c) ESI-Mass (ESI+) spectrum of **NaHL-*S*** and **NaHL-*R*** in methanol. (b) and (d) ESI-Mass (ESI+) spectrum of **3-*S*** and **3-*R*** exists as a single ligand or dual ligand structure in methanol.

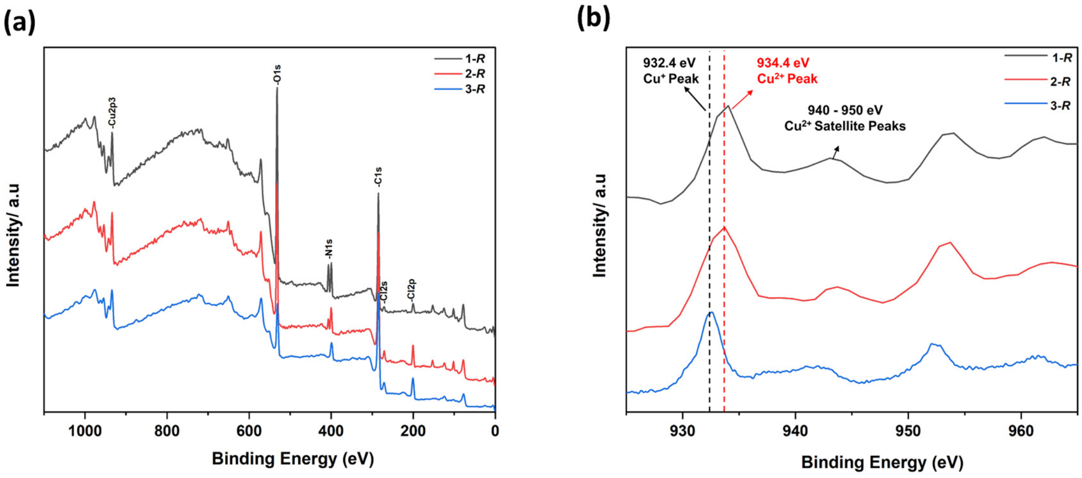

Figure S23 (a) Surveys XPS spectra of **1-*R***, **2-*R*** and **3-*R***; (b) Typical Cu $2p_{3/2}$ XPS spectra for **1-*R***, **2-*R*** and **3-*R***.

Table S1 Crystallographic Data for **1-*R***, **2-*R*** and **3-*R***.

Complex	**1-*R***	**2-*R***	**3-*R***
Formula	$C_{10}H_8ClCuNO_3$	$C_{30}H_{25}Cl_2Cu_2N_4O_6$	$C_{30}H_{27}Cl_2Cu_2N_4O_6$
M(mol^{-1})	289.16	732.91	734.51
T(K)	296(2)	296(2)	296(2)
Crystal system	Orthorhombic	Triclinic	Triclinic
Space group	$P2_12_12_1$	*P1*	*P1*
a (Å)	4.9486(3)	8.0375(14)	8.082(3)
b (Å)	10.8867(7)	10.1831(18)	10.148(4)
a (Å)	19.2189(12)	11.0553(19)	10.966(4)
α (°)	90	66.235(5)	66.893(9)
β (°)	90	79.097(5)	78.699(10)
γ (°)	90	86.761(5)	86.115(11)
V(Å^3)	1035.40(11)	813.0(2)	811.2(5)
Z	4	1	1
ρ(calculate)(g·cm^{-3})	1.855	1.5	1.456
F (000)	580	372	349
2θRange (°)	4.238 - 50.966	4.094 - 57.718	4.108 - 50.906
GOF on F^2	0.985	0.649	0.945
R_{int}	0.1154	0.0186	0.1543
Reflections collected	10343	9925	7082
Independent reflections	1907	8018	5653
$R_1[I > 2\sigma(I)]$	0.0313	0.0523	0.0619
$wR_2[I > 2\sigma(I)]$	0.0648	0.1547	0.1488
R_1(all data)	0.0361	0.107	0.092
wR_2(all data)	0.0668	0.1992	0.1605
Residuals (e Å^{-3})	0.36/-0.34	0.39/-0.55	0.43/-0.57
Flack parameter	0.01(3)	0.29(4)	0.13(5)

Table S2 Cu 2p3/2, Cu 2p1/2 Binding Energies from XPS Measurements

	Cu $2p_{3/2}$ (eV)	Cu $2p_{1/2}$ (eV)
**1-*S***	934.6	954.6
**1-*R***	934.4	954.3
**2-*S***	934	953.6
**2-*R***	934.2	953.9
**3-*S***	932.4	954.2
**3-*R***	932.5	954

Table S3 Selected bond distances (Å) and angles (°) for **1-*S***.

Atom-Atom	Length	Atom-Atom	Length
Cu1-O1	1.879(3)	C10-C7	1.529(5)
Cu1-N1	1.926(3)	C1-C2	1.408(5)
Cu1-O2[1]	1.982(2)	C1-C6	1.423(5)
Cu1-O2	1.988(3)	C7-C9	1.535(5)
Cl1-C3	1.743(4)	C8-C6	1.432(5)
O2-C10	1.303(4)	C3-C2	1.383(5)
O3-C10	1.217(5)	C3-C4	1.381(6)
N1-C8	1.289(5)	C6-C5	1.408(5)
N1-C7	1.467(5)	C10-C7	1.529(5)

[1]-1/2+X,3/2-Y,1-Z

Atom-Atom-Atom	Angle	Atom-Atom-Atom	Angle
O1-Cu1-N1	94.21(12)	O1-C1-C2	117.2(4)
O1-Cu1-O2[1]	89.90(11)	O1-C1-C6	124.8(4)
N1-Cu1-O2[1]	168.90(12)	C2-C1-C6	118.0(3)
O1-Cu1-O2	166.41(12)	N1-C7-C10	108.5(3)
N1-Cu1-O2	83.06(12)	N1-C7-C9	110.8(3)
O21-Cu1-O2[1]	95.25(6)	C10-C7-C9	109.4(3)
C10-O2-Cu1[2]	115.0(2)	N1-C8-C6	125.2(4)
C10-O2-Cu1	115.4(2)	C2-C3-C4	122.4(4)
Cu12-O2-Cu1	128.04(12)	C2-C3-Cl1	117.8(3)
C8-N1-C7	120.0(3)	C4-C3-Cl1	119.9(3)
C8-N1-Cu1	126.1(3)	C3-C2-C1	120.2(4)
C7-N1-Cu1	113.9(2)	C5-C6-C1	119.5(4)
C1-O1-Cu1	127.1(3)	C5-C6-C8	118.0(4)
O3-C10-O2	123.2(4)	C1-C6-C8	122.5(3)
O3-C10-C7	121.0(4)	C5-C4-C3	118.3(4)
O2-C10-C7	115.7(3)	C4-C5-C6	121.6(4)
O1-Cu1-N1	94.21(12)	O1-C1-C2	117.2(4)
O1-Cu1-O21	89.90(11)	O1-C1-C6	124.8(4)

[1]-1/2+X,3/2-Y,1-Z; [2]1/2+X,3/2-Y,1-Z

Table S4. Selected bond distances (Å) and angles (°) for **1-*R***.

Atom-Atom	Length	Atom-Atom	Length
Cu1-O2	1.985(3)	C1-C2	1.415(6)
Cu1-O2[1]	1.985(3)	C1-C6	1.419(7)
Cu1-O1	1.876(3)	C10-C7	1.527(6)
Cu1-N1	1.929(4)	C7-C9	1.534(6)
Cl[1]-C3	1.748(5)	C3-C4	1.374(7)
O2-C10	1.302(5)	C3-C2	1.374(6)
O1-C1	1.309(5)	C8-C6	1.427(6)
O3-C10	1.216(6)	C4-C5	1.376(7)
N1-C7	1.464(6)	C6-C5	1.414(6)
N1-C8	1.294(5)		

[1]-1/2+X,1/2-Y,1-Z

Atom-Atom-Atom	Angle/°	Atom-Atom-Atom	Angle/°
O2[1]-Cu1-O2	95.33(8)	O2-C10-C7	115.6(4)
O1-Cu1-O2[1]	89.84(13)	O3-C10-O2	123.4(4)
O1-Cu1-O2	166.55(14)	O3-C10-C7	120.9(4)
O1-Cu1-N1	94.24(15)	N1-C10-C7	108.6(4)
N1-Cu1-O2	82.99(15)	N1-C7-C9	110.7(4)
N1-Cu1-O2[1]	168.90(15)	C10-C7-C9	109.5(4)
Cu1[2]-O2-Cu1	128.01(16)	C4-C3-Cl1	119.6(4)
C10-O2-Cu1	115.6(3)	C2-C3-Cl1	117.6(4)
C10-O2-Cu1[2]	114.8(3)	C2-C3-C4	122.8(5)
C1-O1-Cu1	127.2(3)	N1-C8-C6	125.1(4)
C7-N1-Cu1	113.9(3)	C3-C4-C5	118.7(5)
C8-N1-Cu1	125.8(3)	C3-C2-C1	119.9(5)
C8-N1-C7	120.3(4)	C1-C6-C8	122.9(4)
O1-C1-C2	117.3(4)	C5-C6-C1	119.5(5)
O1-C1-C6	124.7(4)	C5-C6-C8	117.6(5)
C2-C1-C6	118.0(4)	C4-C5-C6	121.1(5)
O21-Cu1-O2	95.33(8)	O2-C10-C7	115.6(4)

[1]-1/2+X,1/2-Y,1-Z;[2]1/2+X,1/2-Y,1-Z

Table S5 Selected bond distances (Å) and angles (°) for **2-*S*.**

Atom-Atom	Length/Å	Atom-Atom	Length/Å
Cu1-O1	1.922(2)	N1-C7	1.287(4)
Cu1-O2	1.926(2)	N1-C8	1.452(5)
Cu1-N1	1.931(3)	C3-C2	1.398(5)
Cu1-N2	2.026(3)	C3-C4	1.422(5)
Cl1-C1	1.741(4)	C11-C12	1.387(4)
O2-C10	1.269(4)	C4-C5	1.403(5)
O1-C3	1.314(4)	C4-C7	1.434(5)
N2-C15	1.327(4)	C14-C15	1.374(4)
N2-C11	1.342(4)	C2-C1	1.363(5)
C13-C12	1.382(4)	C10-C8	1.525(6)
C13-C14	1.383(5)	C5-C6	1.345(5)
C13-C13[1]	1.485(6)	C1-C6	1.387(5)
O3-C10	1.232(4)	C8-C9	1.442(6)

[1]1-X,2-Y, -Z

Atom-Atom-Atom	Length/Å	Atom-Atom-Atom	Length/Å
O1-Cu1-O2	175.63(10)	N2-Cl1-C12	122.4(3)
O1-Cu1-N1	93.26(12)	C5-C4-C3	118.9(3)
O2-Cu1-N1	83.45(11)	C5-C4-C7	117.8(3)
O1-Cu1-N2	91.71(11)	C3-C4-C7	123.4(3)
O2-Cu1-N2	92.21(11)	C15-C14-C13	120.7(3)
N1-Cu1-N2	164.24(12)	C13-C12-C11	120.4(3)
C10-O2-Cu1	117.5(3)	N2C15-C15	123.1(3)
C3-O1-Cu1	126.2(2)	C1-C2-C3	121.4(4)
C15-N2-Cl1	117.3(3)	O3-C10-O2	125.6(4)
C15-N2-Cu1	121.4(2)	O3-C10-C8	118.3(4)
C11-N2-Cu1	121.3(2)	O2-C10-C8	116.0(3)
C12-C13-C14	116.1(3)	N1-C7-C4	125.3(3)
C12-C13-C13[1]	122.7(4)	C6-C5-C4	122.8(4)
C14-C13-C13[1]	121.2(4)	C2-C1-C6	121.7(4)
C7-N1-C8	119.7(3)	C2-C1-Cl1	118.7(3)
C7-N1-Cu1	125.5(2)	C6-C1-Cl1	119.6(3)
C8-N1-Cu1	113.9(3)	C5-C6-C1	118.1(4)
O1-C3-C2	118.9(3)	C9-C8-N1	115.3(4)
O1-C3-C4	123.9(3)	C9-C8-C10	113.9(4)
C2-C3-C4	117.1(3)	N1-C8-C10	109.1(3)

Table S6 Selected bond distances (Å) and angles (°) for **2-*R***.

Atom-Atom	Length/Å	Atom-Atom	Length/Å
Cu1-O1	1.922(2)	N1-C7	1.287(4)
Cu1-O2	1.926(2)	N1-C8	1.452(5)
Cu1-N1	1.931(3)	C3-C2	1.398(5)
Cu1-N2	2.026(3)	C3-C4	1.422(5)
Cl1-C1	1.741(4)	C11-C12	1.387(4)
O2-C10	1.269(4)	C4-C5	1.403(5)
O1-C3	1.314(4)	C4-C7	1.434(5)
N2-C15	1.327(4)	C14-C15	1.374(4)
N2-C11	1.342(4)	C2-C1	1.363(5)
C13-C12	1.382(4)	C10-C8	1.525(6)
C13-C14	1.383(5)	C5-C6	1.345(5)
C13-C13[1]	1.485(6)	C1-C6	1.387(5)
O3-C10	1.232(4)	C8-C9	1.442(6)

[1]1-X, -Y,2-Z

Atom-Atom-Atom	Length/Å	Atom-Atom-Atom	Length/Å
O1-Cu1-O2	175.63(10)	N2-C11-C12	122.4(3)
O1-Cu1-N1	93.26(12)	C5-C4-C3	118.9(3)
O2-Cu1-N1	83.45(11)	C5-C4-C7	117.8(3)
O1-Cu1-N2	91.71(11)	C3-C4-C7	123.4(3)
O2-Cu1-N2	92.21(11)	C15-C14-C13	120.7(3)
N1-Cu1-N2	164.24(12)	C13-C12-C11	120.4(3)
C10-O2-Cu1	117.5(3)	N2C15-C15	123.1(3)
C3-O1-Cu1	126.2(2)	C1-C2-C3	121.4(4)
C15-N2-C11	117.3(3)	O3-C10-O2	125.6(4)
C15-N2-Cu1	121.4(2)	O3-C10-C8	118.3(4)
C11-N2-Cu1	121.3(2)	O2-C10-C8	116.0(3)
C12-C13-C14	116.1(3)	N1-C7-C4	125.3(3)
C12-C13-C13[1]	122.7(4)	C6-C5-C4	122.8(4)
C14-C13-C13[1]	121.2(4)	C2-C1-C6	121.7(4)
C7-N1-C8	119.7(3)	C2-C1-Cl1	118.7(3)
C7-N1-Cu1	125.5(2)	C6-C1-Cl1	119.6(3)
C8-N1-Cu1	113.9(3)	C5-C6-C1	118.1(4)
O1-C3-C2	118.9(3)	C9-C8-N1	115.3(4)
O1-C3-C4	123.9(3)	C9-C8-C10	113.9(4)
C2-C3-C4	117.1(3)	N1-C8-C10	109.1(3)

[1]1-X, -Y,2-Z

Table S7 Selected bond distances (Å) and angles (°) for **3-*S***.

Atom-Atom	Length/Å	Atom-Atom	Length/Å
Cu1-O1	1.908(9)	C27-28	1.39
Cu1-O2	1.952(8)	C28-C29	1.39
Cu1-N1	2.027(3)	C29-C30	1.39
Cu1-N3	1.928(10)	N3-C7	1.290(15)
Cu2-O3	1.910(8)	N3-C8	1.451(15)
Cu2-O4	1.928(8)	N4-C12	1.515(13)
Cu2-N2	2.017(4)	N4-C14	1.268(14)
Cu2-N4	1.945(7)	C3-C2	1.39
Cl1-C3	1.720(6)	C3-C4	1.39
Cl2-C18	1.720(6)	C2-C1	1.39
O1-C1	1.349(10)	C1-C6	1.39
O2-C10	1.262(14)	C6-C5	1.39
O3-C11	1.282(14)	C6-C7	1.467(13)
O4-C20	1.342(9)	C5-C4	1.39
O5-C10	1.194(15)	C8-C9	1.533(13)
O6-C11	1.281(14)	C8-C10	1.555(15)
N1-C25	1.39	C11-C12	1.506(16)
N1-C21	1.39	C12-C13	1.428(17)
C25-C24	1.39	C14-C15	1.438(13)
C24-C23	1.39	C15-C20	1.39
C23-C22	1.39	C15-C16	1.39
C23-C28	1.540(3)	C20-C19	1.39
C22-C21	1.39	C19-C18	1.39
N2-C26	1.39	C18-C17	1.39
N2-C30	1.39	C17-C16	1.39
C26-C27	1.39	C27-C28	1.39
Cu1-O1	1.908(9)		

Atom-Atom-Atom	Angle/°	Atom-Atom-Atom	Angle/°
O1-Cu1-O2	175.9(4)	C14-N4-Cu2	123.0(7)
O1-Cu1-N1	90.3(3)	C14N4-Cl2	123.4(8)
O1-Cu1-N3	93.3(4)	C2-C3-Cl1	119.5(5)
O2-Cu1-N1	93.2(3)	C2-C3-C4	120
N3-Cu1-O2	84.0(4)	C4-C3-Cl1	120.5(5)
N3-Cu1-N1	164.7(3)	C1-C2-C3	120
O3-Cu2-O4	175.8(4)	O1-C1-C2	117.5(6)
O3-Cu2-N2	91.1(3)	O1-C1-C6	122.5(6)
O3-Cu2-N4	83.3(4)	C6-C1-C2	120
O4-Cu2-N2	93.1(3)	C1-C6-C5	120

O4-Cu2-N4	92.4(3)	C1-C6-C7	126.8(7)
N4-Cu2-N2	168.7(3)	C5-C6-C7	113.2(7)
C1-O1-Cu1	126.0(7)	C4-C5-C6	120
C10-O2-Cu1	117.4(8)	C5-C4-C3	120
C11-O3-Cu2	116.6(7)	N3-C7-C6	120.9(10)
C20-O4-Cu2	125.1(6)	N3-C8-C9	112.4(9)
C25-N1-Cu1	119.0(3)	N3-C8-C10	110.3(9)
C25-N1-C21	120	C9-C8-C10	109.1(9)
C21-N1-Cu1	121.0(3)	O2-C10-C8	114.8(10)
C24-C25-N1	120	O5-C10-O2	126.5(12)
C25-C24-C23	120	O5-C10-C8	118.4(11)
C24-C23-C28	121.3(3)	O3-C11-C12	118.8(9)
C22-C23-C24	120	O6-C11-O3	123.7(12)
C22-C23-C28	118.7(3)	O6-C11-C12	116.6(10)
C21-C22-C23	120	C11-C12-N4	104.7(8)
C22-C21-N1	120	C13-C12-N4	118.0(11)
C26-N2-Cu2	122.6(3)	C13-C12-C11	116.1(11)
C26-N2-C30	120	N4-C14-C15	128.6(9)
C30-N2-Cu2	117.4(3)	C20-C15-C14	121.2(6)
N2-C26-C27	120	C20-C15-C16	120
C26-C27-C28	120	C16-C15-C14	118.3(6)
C27-C28-C23	120.8(3)	O4-C20-C15	124.6(6)
C27-C28-C29	120	O4-C20-C19	115.3(6)
C29-C28-C23	119.1(3)	C19-C20-C15	120
C30-C29-C28	120	C20-C19-C18	120
C29-C30-N2	120	C19-C18-Cl2	119.9(5)
C7-N3-Cu1	128.3(9)	C17-C18-Cl2	120.1(5)
C7-N3-C8	118.2(10)	C17-C18-C19	120
C8-N3-Cu1	113.2(8)	C18-C17-C16	120
C12-N4-Cu2	113.0(7)	C17-C16-C15	120
O1-Cu1-O2	175.9(4)	C14-N4-Cu2	123.0(7)
O1-Cu1-N1	90.3(3)	C14N4-Cl2	123.4(8)
O1-Cu1-N3	93.3(4)	C2-C3-Cl1	119.5(5)
O2-Cu1-N1	93.2(3)	C2-C3-C4	120
N3-Cu1-O2	84.0(4)	C4-C3-Cl1	120.5(5)

Table S8 Selected bond distances (Å) and angles (°) for **3-*R***.

Atom-Atom	Length/Å	Atom-Atom	Length/Å
Cu2-O4	1.942(14)	C28-C27	1.33(3)
Cu2-O3	1.893(16)	C28-C23	1.476(12)
Cu2-N2	2.034(17)	C27-C26	1.42(3)
Cu2-N4	1.957(18)	C25-C24	1.36(3)
Cu1-O1	1.892(16)	C24-C23	1.44(3)
Cu1-O2	1.975(13)	C23-C22	1.42(3)
Cu1-N1	2.011(16)	C22-C21	1.39(3)
Cu1-N3	1.909(18)	C6-C5	1.39
Cl2-C18	1.756(12)	C6-C1	1.39
Cl1-C3	1.698(11)	C6-C7	1.43(3)
O1-C1	1.346(17)	C5-C4	1.39
O2-C10	1.28(3)	C4-C3	1.39
O4-C20	1.353(17)	C3-C2	1.39
O3-C11	1.27(2)	C2-C1	1.39
O5-C10	1.20(3)	C8-C9	1.41(3)
O6-C11	1.24(2)	C8-C10	1.62(3)
N2-C30	1.36(2)	C18-C17	1.39
N2-C26	1.32(3)	C18-19	1.39
N1-C25	1.37(2)	C17-C16	1.39
N1-C21	1.29(3)	C16-C15	1.39
N4-C14	1.26(3)	C15-C20	1.39
N4-C12	1.47(3)	C15-C14	1.45(2)
N3-C7	1.33(3)	C20-C19	1.39
N3-C8	1.45(3)	C12-C11	1.46(3)
C30-C29	1.36(3)	C12-C13	1.55(3)
C29-C28	1.37(3)	C28-C27	1.33(3)
Cu2-O4	1.942(14)		

Atom-Atom-Atom	Angle/°	Atom-Atom-Atom	Angle/°
O4-Cu2-N2	90.6(6)	C21-N1-Cu1	121.1(13)
O4-Cu2-N4	93.7(7)	C21-N1-C25	116.8(18)
O3-Cu2-O4	176.7(8)	C14-N4-Cu2	126.2(17)
O3-Cu2-N2	92.6(7)	C14-N4-Cl2	120(2)
O3-Cu2-N4	83.5(8)	C12-N4-Cu2	111.2(14)
N4-Cu2-N2	166.0(9)	C7-N3-Cu1	122.9(15)
O1-Cu1-O2	175.7(7)	C7-N3-C8	121.4(19)
O1-Cu1-N1	92.9(6)	C8-N3-Cu1	115.7(15)
O1-Cu1-N3	92.3(7)	C29-C30-N2	121.1(19)
O2-Cu1-N1	91.4(7)	C30-C29-C28	119.8(18)
N3-Cu1-O2	83.6(7)	C29-C28-C23	119.7(12)

N3-Cu1-N1	166.9(8)	C27-C28-C29	120.3(18)
C1-O1-Cu1	127.6(11)	C27-C28-C23	119.9(14)
C10-O2-Cu1	116.6(16)	C28-C27-C26	117.9(19)
C20-O4-Cu2	124.3(10)	N2-C26-C27	122.3(19)
C11-O3-Cu2	117.8(14)	C24-C25-N1	121(2)
C30-N2-Cu2	120.4(14)	C25-C24-C23	123(2)
C26-N2-Cu2	121.5(13)	C24-C23-C28	124.3(13)
C26-N2-C30	118.1(18)	C22-C23-C28	123.1(15)
C25-N1-Cu1	122.2(15)	C22-C23-C24	113(2)
O4-Cu2-N2	90.6(6)	C21-N1-Cu1	121.1(13)
O4-Cu2-N4	93.7(7)	C21-N1-C25	116.8(18)
O3-Cu2-O4	176.7(8)	C14-N4-Cu2	126.2(17)
O3-Cu2-N2	92.6(7)	C14-N4-Cl2	120(2)
O3-Cu2-N4	83.5(8)	C12-N4-Cu2	111.2(14)
N4-Cu2-N2	166.0(9)	C7-N3-Cu1	122.9(15)
O1-Cu1-O2	175.7(7)	C7-N3-C8	121.4(19)
O1-Cu1-N1	92.9(6)	C8-N3-Cu1	115.7(15)
O1-Cu1-N3	92.3(7)	C29-C30-N2	121.1(19)
O2-Cu1-N1	91.4(7)	C30-C29-C28	119.8(18)
N3-Cu1-O2	83.6(7)	C29-C28-C23	119.7(12)
N3-Cu1-N1	166.9(8)	C27-C28-C29	120.3(18)
C1-O1-Cu1	127.6(11)	C27-C28-C23	119.9(14)
C10-O2-Cu1	116.6(16)	C28-C27-C26	117.9(19)
C20-O4-Cu2	124.3(10)	N2-C26-C27	122.3(19)
C11-O3-Cu2	117.8(14)	C24-C25-N1	121(2)
C30-N2-Cu2	120.4(14)	C25-C24-C23	123(2)
C26-N2-Cu2	121.5(13)	C24-C23-C28	124.3(13)
C26-N2-C30	118.1(18)	C22-C23-C28	123.1(15)
C25-N1-Cu1	122.2(15)	C22-C23-C24	113(2)
O4-Cu2-N2	90.6(6)	C21-N1-Cu1	121.1(13)
O4-Cu2-N4	93.7(7)	C21-N1-C25	116.8(18)
O3-Cu2-O4	176.7(8)	C14-N4-Cu2	126.2(17)
O3-Cu2-N2	92.6(7)	C14-N4-Cl2	120(2)
O3-Cu2-N4	83.5(8)	C12-N4-Cu2	111.2(14)
